# Predicting patient-related outcomes after atrial fibrillation ablation: insights from explainable artificial intelligence and digital health

**DOI:** 10.1093/ehjdh/ztaf090

**Published:** 2025-08-07

**Authors:** Rafael Silva-Teixeira, João Almeida, Francisco A Caramelo, Paulo Fonseca, Marco Oliveira, Helena Gonçalves, João Primo, Ricardo Fontes-Carvalho

**Affiliations:** Department of Cardiology, Gaia Hospital Center, Rua Conceição Fernandes, 4434-502 Vila Nova de Gaia, Portugal; Center for Innovative Biomedicine and Biotechnology (CIBB), University of Coimbra, Azinhaga de Santa Comba, Celas, 3000-548 Coimbra, Portugal; Clinical Academic Center of Coimbra (CACC), Praceta Professor Mota Pinto, Celas, 3004-561 Coimbra, Portugal; Faculty of Medicine, Coimbra Institute for Clinical and Biomedical Research (iCBR) Area of Environment Genetics and Oncobiology (CIMAGO), Institute of Biophysics, University of Coimbra, Azinhaga de Santa Comba, Celas 3000-548, Portugal; Department of Cardiology, Gaia Hospital Center, Rua Conceição Fernandes, 4434-502 Vila Nova de Gaia, Portugal; Center for Innovative Biomedicine and Biotechnology (CIBB), University of Coimbra, Azinhaga de Santa Comba, Celas, 3000-548 Coimbra, Portugal; Clinical Academic Center of Coimbra (CACC), Praceta Professor Mota Pinto, Celas, 3004-561 Coimbra, Portugal; Faculty of Medicine, Coimbra Institute for Clinical and Biomedical Research (iCBR) Area of Environment Genetics and Oncobiology (CIMAGO), Institute of Biophysics, University of Coimbra, Azinhaga de Santa Comba, Celas 3000-548, Portugal; Department of Cardiology, Gaia Hospital Center, Rua Conceição Fernandes, 4434-502 Vila Nova de Gaia, Portugal; Department of Cardiology, Gaia Hospital Center, Rua Conceição Fernandes, 4434-502 Vila Nova de Gaia, Portugal; Department of Cardiology, Gaia Hospital Center, Rua Conceição Fernandes, 4434-502 Vila Nova de Gaia, Portugal; Department of Cardiology, Gaia Hospital Center, Rua Conceição Fernandes, 4434-502 Vila Nova de Gaia, Portugal; Department of Cardiology, Gaia Hospital Center, Rua Conceição Fernandes, 4434-502 Vila Nova de Gaia, Portugal; Department of Surgery and Physiology, Faculty of Medicine of the University of Porto, Alameda Professor Hernâni Monteiro 4200-319 Porto, Portugal; Cardiovascular R&D Centre-UnIC@RISE, Faculty of Medicine of the University of Porto, Alameda Professor Hernâni Monteiro, 4200-319 Porto, Portugal

**Keywords:** Atrial Fibrillation, Quality of Life, Patient-Reported Outcomes, Catheter Ablation, Machine Learning, Digital Health

## Abstract

**Aims:**

Quality of life (QoL) improvement is a primary driver for atrial fibrillation (AF) catheter ablation (CA), yet its determinants remain unclear. We aimed to identify patient phenotypes with distinct post-ablation QoL trajectories, determine their key predictors, and clarify their association with arrhythmia recurrence and reintervention.

**Methods and results:**

We prospectively followed 213 patients (median age 60 years, 31% female) undergoing AF CA at a tertiary hospital for 2.2 years [interquartile range (IQR): 1.6–2.6]. A digital health application collected real-time electronic patient-reported outcomes (PROs), including the AF Effect on QoL (AFEQT) questionnaire. Reference charts were generated from QoL trajectories of recurrence-free patients. Machine learning (ML) identified subgroups with distinct QoL trajectories, and explainable artificial intelligence (AI) highlighted key predictors. Quality of life improved by +26 AFEQT points [95% confidence interval (CI): 18–33] within 3 months post-ablation and remained stable thereafter, despite significant heterogeneity in individual responses. Patients with AF recurrence showed significantly lower QoL gains (*P* = 0.010). Machine learning identified three phenotypes: a younger cluster with the largest QoL improvements, an emotive cluster with higher recurrence rates and minimal QoL benefits despite additional antiarrhythmic reinterventions, and an older cluster with established cardiovascular risk factors. Anxiety, age, and AF duration emerged as key discriminators.

**Conclusion:**

ML defined three clinically coherent phenotypes, each exhibiting distinct QoL trajectories and ablation outcomes. Explainable AI clarified how individual psychological and biological traits interact to shape these outcomes, highlighting the potential for tailored multidisciplinary care beyond individualized rhythm control strategies.

## Graphical Abstract

**Graphical Abstract:**
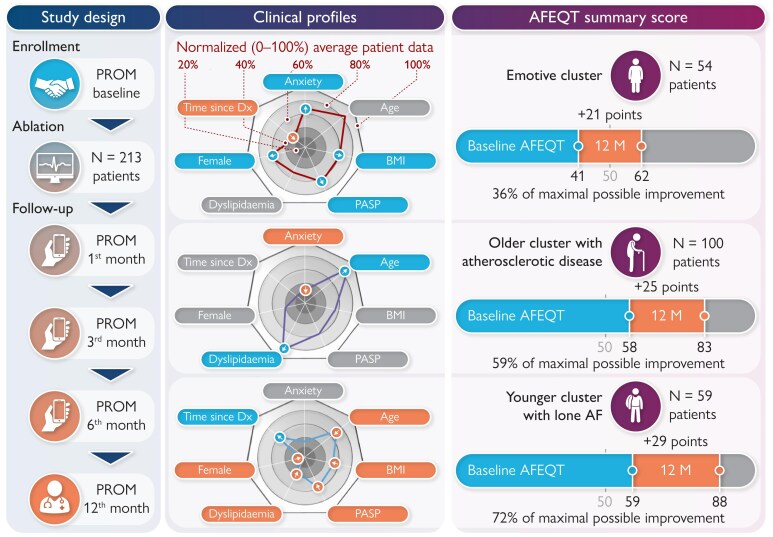
Clinical phenotypes and associated outcomes. Electronic patient-reported outcomes measurements from patients undergoing their first AF ablation were prospectively collected through a digital health app. By applying machine learning and explainable artificial intelligence techniques, we identified three distinct clinical profiles based on the interaction of clinical characteristics and outcomes. AF, atrial fibrillation; Dx, diagnosis.

Key learning points

1. **What is already known**
  - The primary goals of atrial fibrillation (AF) ablation are symptom alleviation and quality of life (QoL) improvement.
  - Ablation success is typically measured by AF recurrence, even though QoL can still improve despite recurrent episodes.
  - Prognostic research in AF ablation predominantly targets predictors of arrhythmia recurrence, frequently overlooking direct drivers of QoL improvement and the complex interplay of diverse patient attributes.
2. **What this study adds**
  - This study applied explainable machine learning to identify three clinically coherent patient phenotypes (younger, older, and emotive clusters), each characterized by distinct longitudinal QoL trajectories following AF ablation.
  - Key clinical and psychological factors (notably anxiety levels, age, and AF duration) were pinpointed as primary discriminators distinguishing these patient phenotypes.
  - The characterization of these phenotypes elucidates how psychological and biological traits interact to shape post-ablation QoL and clinical outcomes, underscoring the need for tailored, multidisciplinary management strategies that extend beyond conventional rhythm control to optimize overall patient well-being.

## Introduction

Atrial fibrillation (AF), the most common sustained arrhythmia, significantly impacts morbidity, mortality, and quality of life (QoL).^1^ Catheter ablation (CA) is a rhythm control strategy primarily aimed at alleviating symptoms and enhancing QoL. Nevertheless, procedural success continues to be judged almost exclusively by freedom from recurrent arrhythmia. This surrogate endpoint correlates poorly with symptom burden and cardiovascular outcomes, and systematically underestimates the clinical benefit of ablation.^2^ Accordingly, most prognostic research has focused on predicting AF recurrence rather than uncovering the key drivers of QoL gains, yielding predictive models with limited value for optimizing patient selection or maximizing QoL benefits.

Patient-reported outcomes (PROs) provide the most direct measure of the treatment benefit perceived by patients and are increasingly incorporated in clinical trials, yet they remain infrequently used in routine clinical practice. Evidence about determinants of AF-related QoL is largely derived from cross-sectional studies in ablation-naïve cohorts.^3–5^ Prospective studies suggest that, after ablation, many baseline factors that differentiate QoL in untreated patients no longer discriminate between individuals who do or do not achieve durable QoL gains.^6,7^ Moreover, since patients often carry several comorbidities, it is likely that QoL disparities arise from multiple interacting factors rather than any single variable.^8^

Cluster analysis, an unsupervised machine-learning (ML) technique, has been employed to group patients with similar comorbidity profiles across multiple correlated covariates.^9^ However, reproducibility of these clusters has been limited, largely owing to heterogeneity in variable selection, cohort characteristics, and clustering algorithms.^9,10^ Bayesian profile regression may refine patient grouping by integrating PROs into the clustering process.^11^ These ML models may be leveraged by recent digital health technologies, particularly electronic PRO (ePRO) platforms, that enable detailed and real-time tracking of QoL.^12^

In this study, we aimed to identify patient phenotypes with distinct post-ablation QoL trajectories, determine the key predictors differentiating those phenotypes, and clarify their association with arrhythmia recurrence and reintervention.

## Methods

### Study design

This prospective cohort study enrolled patients with recurrent symptomatic AF scheduled for a first CA at a tertiary hospital between 2021 and 2022.

Following AF ablation, a comprehensive follow-up (FUP) programme was implemented (see Supplementary material online, *Table S1*), which included scheduled visits, teleconsultations, and remote monitoring using a digital health platform (Promptly Health^®^). The programme featured: (i) a web-based interface for professionals to record clinical outcomes and access ePROs and (ii) a mobile application for patients to report symptoms, vital signs, and complete questionnaires (see Supplementary material online, *Figure S1*). This study was approved by the local Ethics Committee and is in accordance with the principles of the Helsinki Declaration.

### Patient-reported outcomes

The AF Effect on QoL (AFEQT) questionnaire was used to assess AF-specific QoL. This questionnaire is a validated instrument comprising items across three domains: symptoms, daily activities, and treatment concerns.^13^ The overall score ranges from 0 to 100, with scores below 60 typically representing moderate to severe impairment.^14^ AFEQT overall and subdomain scores were analysed longitudinally using a mixed-effects model, which included random intercepts and slopes, accommodating for individual differences in baseline scores and trajectories. Natural cubic splines were added to the model to account for potential non-linear temporal trends (see Supplementary Methods). A sensitivity analysis assessed how robust statistical inferences were to possible violations of missing at random assumptions in longitudinal AFEQT questionnaire data (see Supplementary Methods). To examine the relationship between QoL and procedural outcomes, AFEQT scores were standardized by generating normative reference curves and time-adjusted *Z*-scores derived from patients without AF recurrence. The *Z*-scores of patients with recurrence were then compared against these reference curves to detect significant deviations (see Supplementary Methods). Patients who completed the AFEQT questionnaire on fewer than two occasions during the 12-month follow-up were classified as non-responders and compared with responders to evaluate potential response bias.

Emotional and mental well-being were assessed using PROMIS^®^ Anxiety and Depression short forms.

Questionnaire completeness rate, defined as the proportion of completed vs. assigned questionnaires, was assessed at both patient-specific and scheduled time points.

### Semisupervised machine learning to determine phenogroups

#### Data preprocessing

To address missing data in both baseline characteristics (see Supplementary material online, *Table S2*) and longitudinal outcomes (see Supplementary material online, *Table S3*), we combined multilevel joint modelling with multiple imputation. This method integrates the previously defined AFEQT longitudinal model with the joint distribution of baseline covariates, ensuring that the imputation process considers the relationships between the baseline variables and the outcome trajectory of each individual. Using the *jomo* R package, 20 imputed datasets were generated. Next, we applied principal component analysis to reduce redundancy in echocardiographic variables (see Supplementary Methods). Correlated variables were transformed into five uncorrelated principal components based on the number of components with eigenvalues >1 and variance explained >50% (see Supplementary material online, *Figure S2*).

#### Clustering analysis

Profile regression mixture modelling was used for patient grouping using the *PReMiuM* R package. This semisupervised model incorporates outcomes into the clustering process, aiming to identify latent subgroups that differ not only in covariates but also in outcome patterns. Demographic, clinical and psychometric data, and principal components previously derived from echocardiographic variables were used as inputs (detailed in Supplementary Methods). The AFEQT summary score at month 12, adjusted for the baseline score, was entered as the outcome variable. Clustering results from multiple imputed datasets were aggregated, and consensus clustering was applied to ensure the stability and reproducibility of the identified clusters (see Supplementary Methods). The cluster labels were then used as target class labels for supervised learning.

### Supervised machine learning to distinguish each phenogroup

Clustering analysis identifies homogeneous subgroups within a heterogeneous population, but it does not inherently explain why individuals are assigned to specific clusters. To address these limitations, we developed a *post-hoc* supervised classifier that learns to uncover patterns within clinical baseline data that can predict cluster assignment. XGBOOST was chosen for its ability to capture complex interactions and non-linearities among variables. To reduce overfitting, highly correlated variables were removed, and a nested five-fold cross-validation scheme was applied (see Supplementary Methods). To address class imbalances, higher weights were assigned to underrepresented classes, and the weighted F1-score was used as the evaluation metric. To understand how each feature influences cluster membership, we used SHapley Additive exPlanations (SHAP). By decomposing the model's predictions into additive contributions from each feature, SHAP reveals the magnitude and direction of their impact on cluster assignment.^15^

### Statistical analysis

Categorical variables were expressed as absolute and relative frequencies, while continuous variables were reported as medians and interquartile ranges (IQRs) or 95% confidence intervals (CIs). To compare continuous variables between clusters, we pooled mean differences across 1000 bootstrapped samples for each imputed dataset and used Wald statistics in accordance with Rubin's rules. For categorical variables, we combined χ^2^ statistics using Rubin's rules. The Bonferroni correction was applied for multiple comparisons.

### Clinical outcomes analysis across the phenotypic groups

The endpoint of recurrence was defined as any recurrence of AF or atrial tachycardia (AT) lasting >30 s documented by electrocardiographic tracing, that occurred after AF ablation, including a blanking period of 8 weeks. Late recurrence was defined as any recurrence after the blanking period. Additional endpoints included time to an antiarrhythmic reintervention, time to the first emergency department visit due to AF, and the first major adverse cardiovascular event (MACE). Antiarrhythmic reintervention was defined as the reintroduction of antiarrhythmic medication, a new ablation procedure, or electrical cardioversion. MACE was defined as a composite of cardiovascular death, non-fatal myocardial infarction, non-fatal stroke, and acute heart failure requiring hospitalization. Log-rank tests were used to compare the incidence of each outcome across the identified clusters.

### Statistical software

All analyses and graphs were performed using R version 4.3.2 and Python version 3.11.8. Two-sided *P* < 0.05 was considered statistically significant.

## Results

### Study population

Of the 277 patients undergoing AF ablation, 23 were excluded for not consenting to the digital app, 18 for follow-up non-compliance, and 23 for answering only one questionnaire (non-responders) (see Supplementary material online, *Figure S3*). We found no significant baseline differences between responders and non-responders (see Supplementary material online, *Table S4*). The final cohort comprised 213 patients, with a median age of 60 years (IQR: 51–67), 31% were women, and the median CHA_2_DS_2_-VASc score was 1 (IQR: 0–2). The median AF duration was 2.5 years (IQR: 1.5–5.5), and 81% of the patients had paroxysmal AF (*Table 1*). The median follow-up period (until the last update on 20 December 2023) was 26 months (IQR: 19–31). During follow-up, 280 of 330 (85%) scheduled in-person consultations and 722 of 809 (89%) teleconsultations were completed.

**Table 1:** Baseline characteristics of the population

	Population (*N* = 213)
Demographics	
Age (years)	60 (51, 67)
Female	65 (31)
BMI (kg/m^2^)	27.1 (24.8, 29.4)
Arrhythmia	
Atrial fibrillation	
Paroxysmal	173 (81)
Persistent	40 (19)
CHA_2_DS_2_-VASc	
0–1 (Lowest risk)	129 (57)
2	50 (23)
3	33 (15)
≥4 (Highest risk)	10 (5)
Years since onset of AF	2.5 (1.5, 5.5)
No prior direct cardioversion	79 (44)
History of atrial flutter	38 (18)
Prior atrial flutter ablation	3 (1)
Antiarrhythmic drugs	131 (62)
Beta-blockers	147 (69)
Procedure	
Energy	
Crioenergy	48 (23)
Radiofrequency	165 (77)
Cavo-tricuspid isthmus ablation	30 (14)
Posterior wall ablation	21 (10)
Comorbidities	
Thyroid dysfunction	24 (11)
History of smoking	49 (23)
Dyslipidaemia	107 (50)
Heart failure	21 (10)
Arterial hypertension	100 (47)
Diabetes	19 (9)
Cerebrovascular disease	13 (6)
Coronary artery disease	8 (4)
Echocardiographic	
LA volume index (mL/m^2^)	37 (31, 45)
LV ejection fraction (%)	60 (56, 63)
RWT	0.38 (0.33, 0.42)
LV mass index (mg/m^2^)	90 (76, 102)
sPAP	23 (20, 25)
eGFR (mL/min/1.73 m^2^)	87 (77, 98)
Haemoglobin (g/dL)	14.7 (13.5, 15.4)

Quantitative variables: median (p25, p75); Categorical variables: *N* (frequency, %).

AF, atrial fibrillation; BMI, body mass index; eGFR, estimated glomerular filtration rate; LA, left atrium; LV, left ventricle; RWT, relative wall thickness; sPAP, systolic pulmonary artery pressure.

### Longitudinal change in atrial fibrillation-related quality of life after catheter ablation

The completion rate of the AFEQT questionnaire was 70%, starting at 49.3% at baseline and increasing over time. The analysis of the missing AFEQT longitudinal data showed no identifiable pattern, indicating that the data were missing at random (see Supplementary material online, *Table S5*, Supplementary Results).

**Figure 1:**
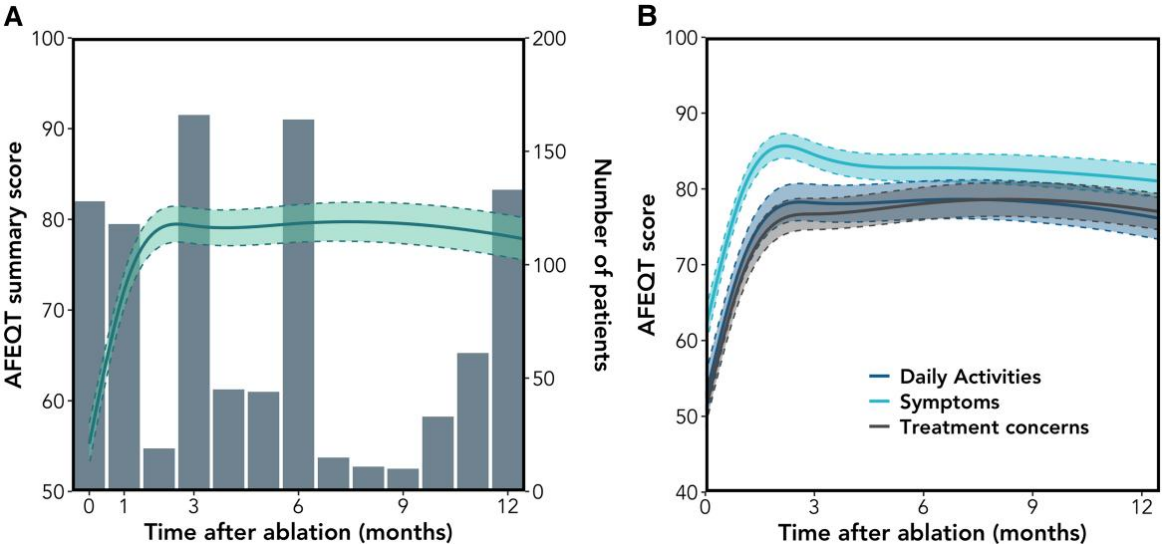
Longitudinal atrial fibrillation effect on QualiTy-of-life overall and subdomain scores. Atrial fibrillation effect on QualiTy-of-life (*A*) overall summary and (*B*) subdomains scores (solid line) with 95% confidence interval (shaded area) modelled as a non-linear function of time. (*A*) Vertical bars indicate the number of patients who responded within each time frame, expressed on the right vertical axis. AF, atrial fibrillation.

**Figure 2:**
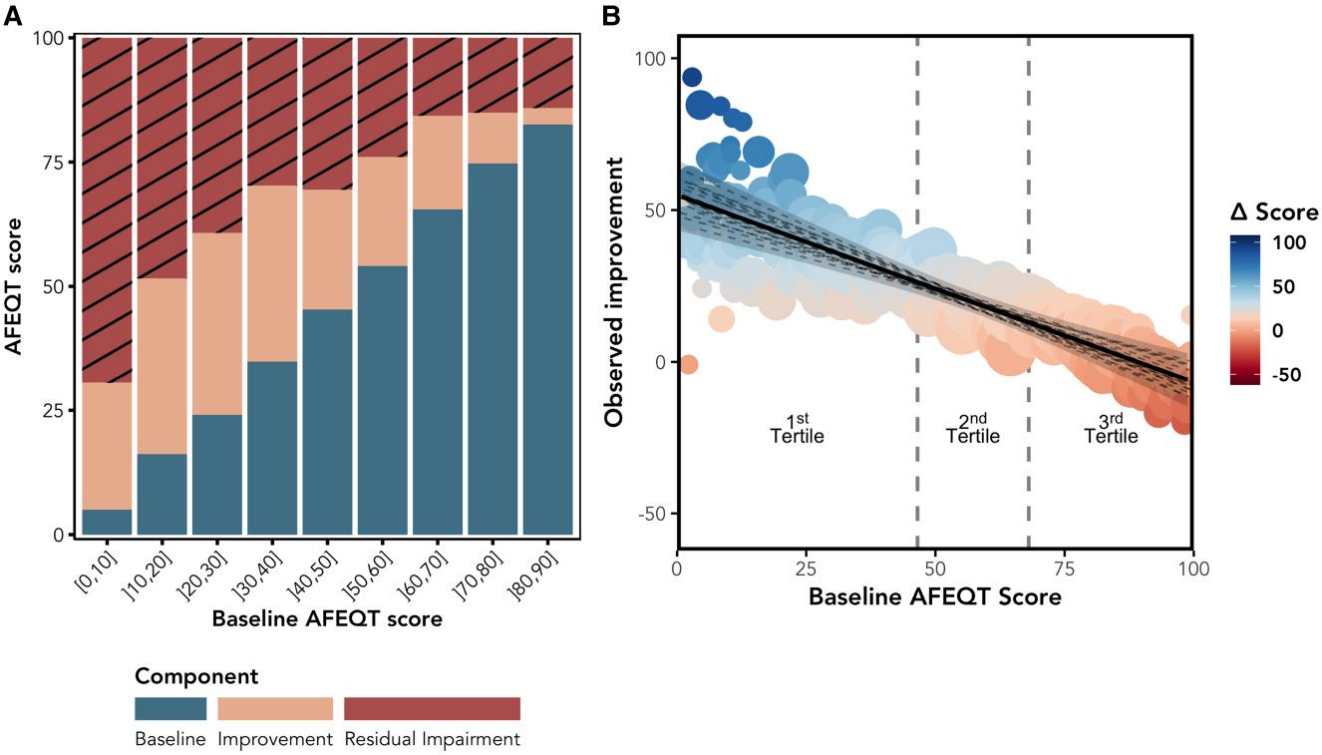
Improvement in the atrial fibrillation effect on QualiTy-of-life score. Changes in the atrial fibrillation effect on QualiTy-of-life summary score by month 12 according to the baseline score. (*A*) Each stacked bar represents the final 12-month score decomposed into three segments: the baseline value (blue/ lower segment), the absolute improvement achieved by month 12 (orange/ middle segment), and the remaining residual impairment (striped red/ upper hatched segment), defined as the difference between the 12-month score and the maximum possible value of 100. Lower baseline scores generally correspond to larger absolute improvements, yet a portion of residual impairment persists across all baseline severity categories. (*B*) There is a moderate association between baseline atrial fibrillation effect on QualiTy-of-life score and observed improvement. The solid line represents the pooled estimated from multiple imputations (dashed black lines) and estimated 95% confidence interval (shaded grey area). Every 10-point increase in the baseline atrial fibrillation effect on QualiTy-of-life score was associated with a 6.2-point decrease in the observed improvement (CI: 4.23–8.11; *P* < 0.001; *R*^2^ = 39%).

The mean AFEQT overall score was 51 ± 27 at baseline (*Table 2*; Supplementary material online, *Figure S4*) and substantially improved by 1-month post-ablation (+17 points, CI: 11–24; *P* < 0.001), peaked at 3 months (+26 points, CI: 18–33; *P* < 0.001), and remained stable thereafter (*Figure 1A*). All AFEQT subdomains followed a similar pattern (*Figure 1B*). One year after AF ablation, patients continued to exhibit a median 50% (IQR: 20–75%) of their initial QoL impairment (calculated as 100—baseline AFEQT score; *Figure 2A*).

**Table 2:** AFEQT summary score across follow-up

Follow-up point	Questionnaire completion rate (%)	Median (25th, 75th percentile)	Estimated mean (SD)	Mean adjusted difference (95% CI)	*P*-value
Baseline	105 (49.3)	52 (38, 68)	51 (27)	—	
1 month	142 (66.7)	75 (55, 88)	68 (21)	17 (11, 24)	<0.001
3 months	176 (82.6)	82 (66, 90)	77 (19)	26 (18, 33)	<0.001
6 months	197 (92.5)	80 (65, 92)	77 (19)	27 (20, 33)	<0.001
12 months	164 (77.0)	81 (66, 93)	76 (22)	25 (18, 32)	<0.001

Questionnaire completion rate indicates the number of patients (and proportion of the total cohort) who completed the AFEQT questionnaire at each scheduled timepoint. Score distributions are presented as both median (interquartile range) and model-estimated mean (standard deviation). Mean adjusted difference quantifies least-squares estimates of change from baseline with corresponding 95% CI, derived from mixed-effects modelling that accounts for within-subject correlation. *P*-values indicate the statistical significance of temporal changes compared with baseline measurements.

CI, confidence interval; SD, standard deviation.

Patients with higher baseline AFEQT scores experienced less improvement compared with those with lower baseline scores, as shown in *Figure 2B*. Every 10-point increase in the baseline AFEQT score was associated with a 6.2-point decrease in observed improvement (CI: 4.23–8.11; *P* < 0.001).

**Figure 3:**
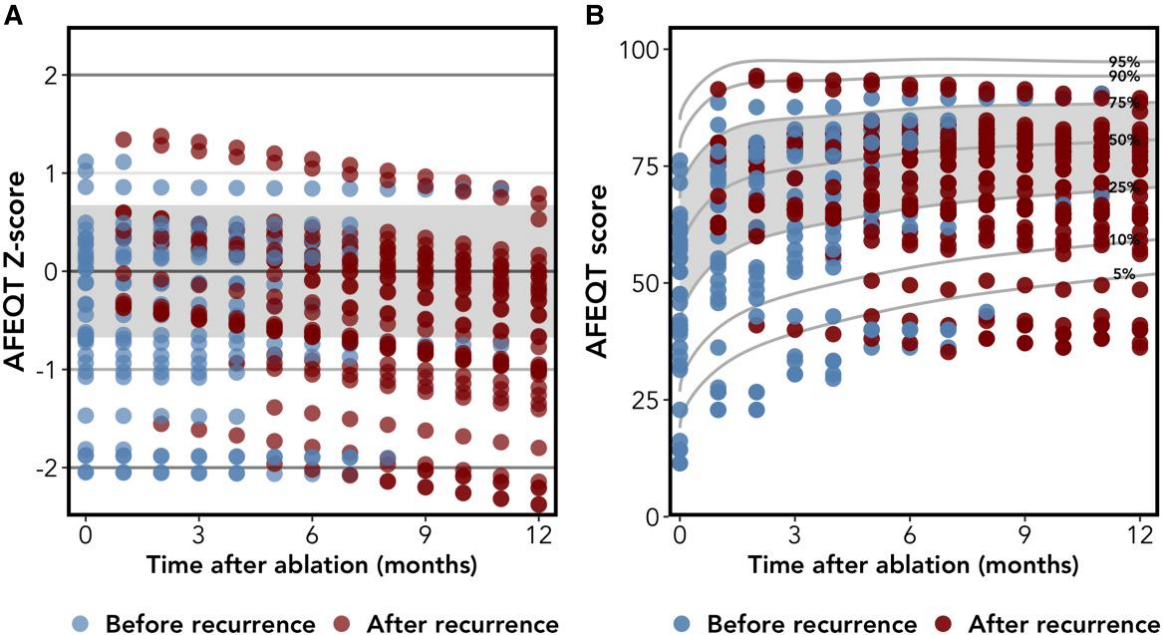
Influence of recurrence on the atrial fibrillation effect on QualiTy-of-life score. These graphs show atrial fibrillation effect on QualiTy-of-life *Z*-scores (*A*) and percentiles (*B*) over 12 months post-ablation. Reference lines were generated using data from patients without atrial fibrillation recurrences during follow-up. Atrial fibrillation effect on QualiTy-of-life results from patients who relapsed were mapped onto these curves and represented by dots. Blue dots denote the atrial fibrillation effect on QualiTy-of-life score prior to recurrence, while red dots indicate the score after recurrence.

Patients experiencing AF recurrence demonstrated significantly attenuated QoL improvement compared with recurrence-free counterparts. Mapping recurrent patients onto reference curves from recurrence-free counterparts showed a consistent downward shift in both *Z*-scores (*Figure 3A*) and percentiles (*Figure 3B*). A significant interaction between time and recurrence status was found (mean monthly *Z*-score change: −0.05 ± 0.02; *P* = 0.010).

### Identification of clinical phenotypes using explainable artificial intelligence

A semisupervised clustering model identified three distinct clinical phenotypes that encompassed approximately 28, 47, and 25% of the population (see Supplementary material online, *Figure S5*). We then used these phenotypes as target labels for a supervised classifier, which achieved an area under the receiver-operating characteristic curve of 0.95 (sensitivity 86%, specificity 89%) with high classification accuracy (weighted F1-score of 0.85; Supplementary material online, *Table S6*).

**Figure 4:**
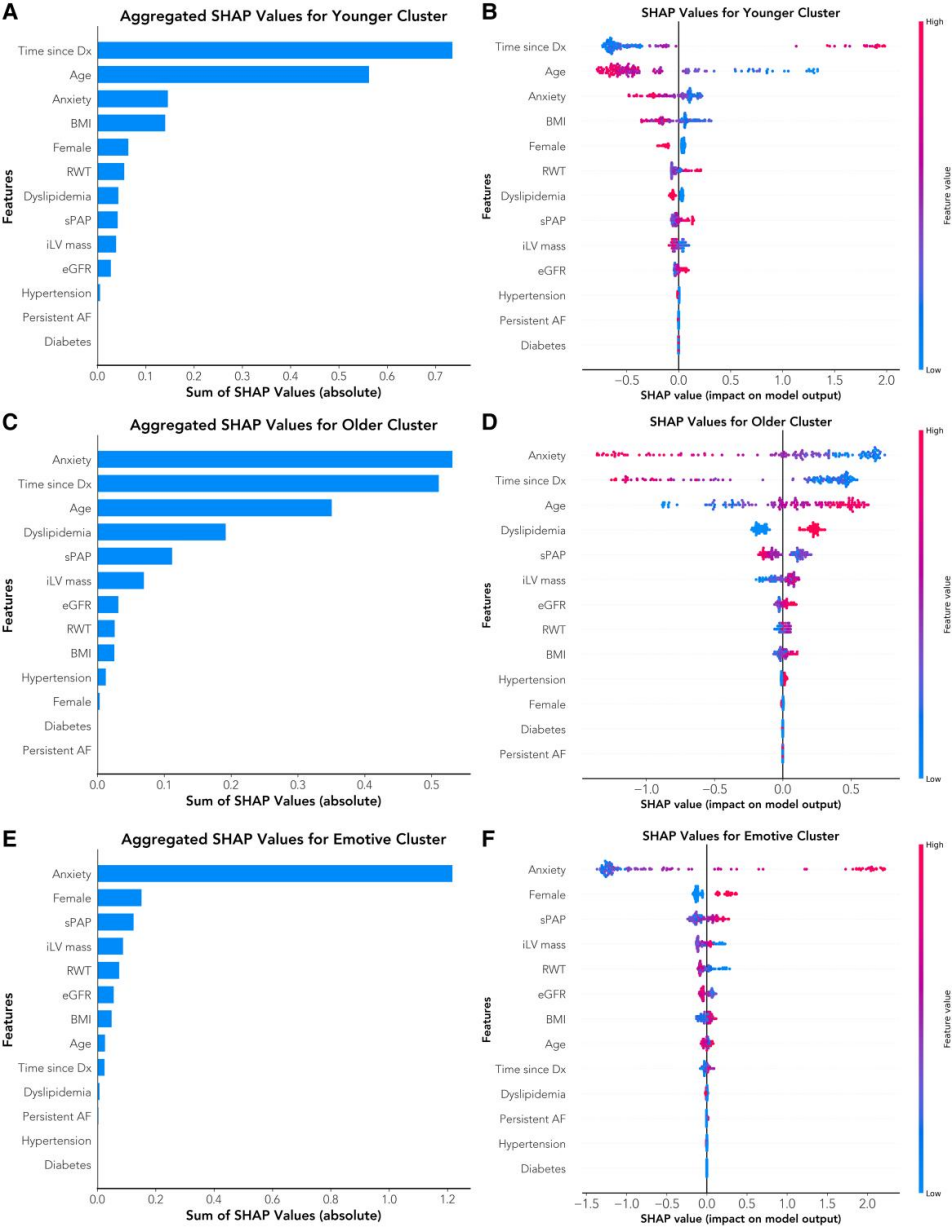
Global explanations of features in our explainable machine learning model. (*A*, *C*, *E*) The aggregated bar plots show the mean absolute SHAP (SHapley Additive exPlanations) values for each predictor, thus ranking variables by their overall impact on assigning patients to a specific cluster. A longer bar implies a stronger average effect in driving classification towards that cluster. (*B*, *D*, *F*) In the bee swarm plots, each row corresponds to a feature, and each dot represents a single patient. Overlapping points are jittered in the *y*-axis direction, so we get a sense of the distribution of the Shapley values per feature. The *x*-axis is the SHAP value for that feature, which can be positive or negative. Positive SHAP values push the predicted class towards that cluster, while negative values push it away. The colour gradient reflects the raw value of that feature (for example, blue for a lower value and pink for a higher value). AF, atrial fibrillation; BMI, body mass index; CI, confidence interval; Dx, diagnosis; eGFR, estimated glomerular filtration rate; iLV mass, indexed left ventricle mass; RWT, relative wall thickness; sPAP, systolic pulmonary artery pressure.

**Figure 5:**
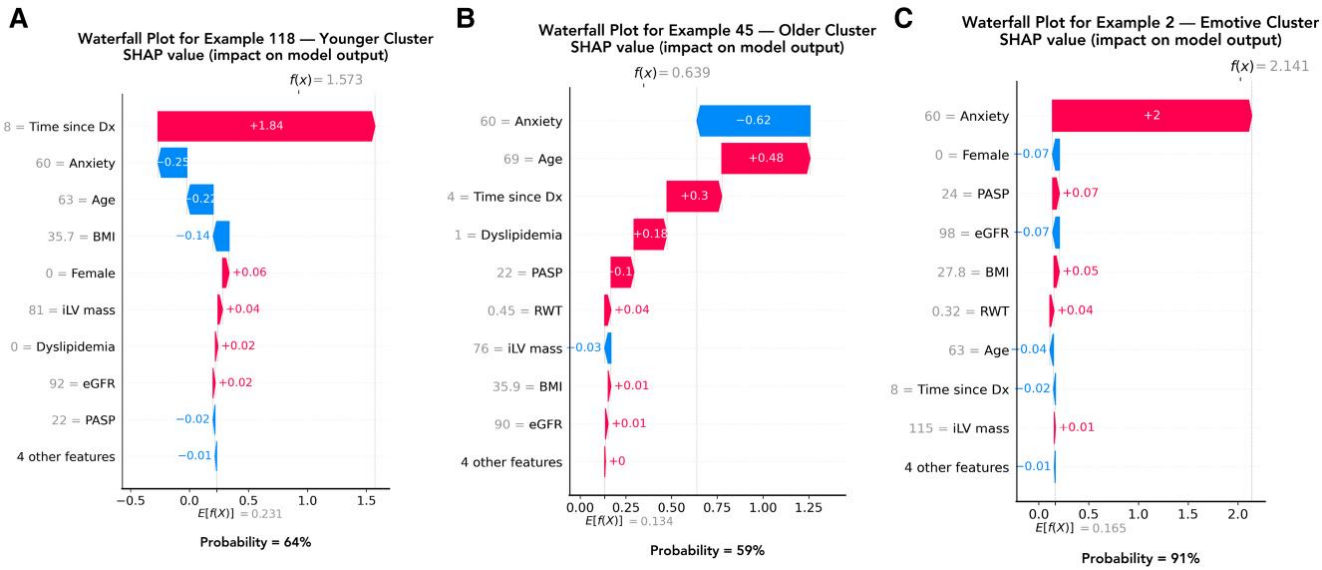
Waterfall plots for a random patient from each phenogroup. The waterfall plots display SHAP (SHapley Additive exPlanations) values in increasing order of magnitude, with red indicating features that increase risk and blue indicating features that decrease the probability of cluster membership (*A*, *B*, *C*). The prediction starts from the baseline. The baseline for Shapley values is the average of all predictions. Each feature's SHAP value is added to determine cluster membership probability. Abbreviations are the same as in *Figure 4*.

*Table 3* summarizes the baseline characteristics of the three phenotypes. *Figure 4* depicts the top defining features for each cluster in descending order, as evaluated by average absolute SHAP values. Globally, anxiety, time since diagnosis, age, female sex, dyslipidaemia, body mass index (BMI), and echocardiographic variables [systolic pulmonary artery pressure (sPAP), left ventricular (LV) mass, and relative wall thickness (RWT)] were the key drivers of cluster assignment (*Figure 4A, C, E*). In contrast, AF temporal classification (paroxysmal vs. persistent) and the left atrial (LA) volume index (LAVI) did not strongly discriminate among the clusters. Depression was excluded during feature selection because of its high collinearity with anxiety. Bee swarm plots further illustrated the direction of each feature's influence on the probability of cluster membership (*Figure 4B, D, F*). Based on the most influential features and their effects, we labelled the clusters as follows: (i) younger cluster (characterized by younger age, fewer comorbidities, and longer time to ablation since AF diagnosis); (ii) older cluster (associated with low reported anxiety, recently diagnosed AF, older age, a higher burden of traditional cardiovascular risk factors and signs of LV hypertrophy); and (iii) emotive cluster (marked by high baseline anxiety, predominantly female sex, and elevated sPAP with minimal LV remodelling; *Central Illustration*). Waterfall plots (*Figure 5*) provided personalized feature attributions for a representative patient from each cluster, demonstrating how individual feature values combine to predict the probability of cluster membership. Importantly, the same feature value (e.g. anxiety) can have varying effects across individuals, depending on its interplay with other clinical characteristics.

**Table 3:** Comparison of clinical characteristics across clinical phenotypes

	Younger cluster/lone AF (*N* = 59)	Older atherosclerotic cluster (*N* = 100)	Emotive cluster (*N* = 54)	*P*-value
Demographics				
Age (years)	52 (44, 59)	64 (57, 68)	60 (51, 66)	<0.001
Female	5 (8)	29 (29)	31 (57)	<0.001
BMI (kg/m^2^)	26.3 (24.8, 27.8)	27.1 (24.6, 29.4)	28.0 (25.0, 31.8)	<0.001
Arrhythmia				
Atrial fibrillation				0.270
Paroxysmal	52 (88)	78 (78)	43 (80)	
Persistent	7 (12)	22 (22)	11 (20)	
CHA_2_DS_2_-VASc				<0.001
0–1 (Lowest risk)	49 (84)	43 (43)	28 (51)	
2	7 (12)	31 (31)	12 (22)	
3	3 (5)	21 (21)	9 (17)	
≥4 (Highest risk)	0 (0)	5 (5)	5 (9)	
Years since onset of AF	7.5 (2.6, 10.1)	2.5 (1.5, 3.5)	2.5 (1.5, 4.5)	<0.001
History of atrial flutter	7 (12)	20 (20)	11 (20)	0.369
Antiarrhythmic drugs	36 (61)	61 (61)	34 (63)	0.968
Beta-blockers	39 (66)	67 (67)	41 (76)	0.443
Procedure				
Energy				0.222
Crioenergy	18 (31)	19 (19)	11 (20)	
Radiofrequency	41 (69)	81 (81)	43 (80)	
Cavo-tricuspid isthmus ablation	4 (7)	18 (18)	8 (15)	0.143
Posterior wall ablation	5 (8)	11 (11)	5 (9)	0.863
Comorbidities				
Thyroid dysfunction	4 (7)	10 (10)	10 (19)	0.123
History of smoking	12 (20)	27 (27)	10 (19)	0.417
Dyslipidaemia	15 (25)	72 (72)	20 (37)	<0.001
Heart failure	6 (10)	8 (8)	7 (13)	0.612
Arterial hypertension	16 (27)	57 (57)	27 (50)	0.001
Diabetes	4 (7)	14 (14)	1 (2)	0.029
Cerebrovascular disease	3 (5)	5 (5)	5 (9)	0.498
Coronary artery disease	0 (0)	6 (6)	2 (4)	0.144
Echocardiographic				
LA volume index (mL/m^2^)	35 (28, 39)	39 (33, 45)	40 (34, 50)	<0.001
LV ejection fraction (%)	60 (57, 63)	60 (57, 62)	59 (56, 63)	0.001
Indexed LV mass (g/m^2^)	87.6 (77.8, 98.8)	91.5 (81.3, 104.0)	84.45 (68.7, 100.7)	<0.001
RWT	0.37 (0.33, 0.44)	0.39 (0.35, 0.43)	0.37 (0.33, 0.42)	0.049
sPAP (mmHg)	21 (18, 25)	22 (19, 25)	24 (21, 28)	0.004
PROMIS® anxiety, *T*-score	47 (42, 51)	47 (42, 51)	62 (58, 65)	<0.001
PROMIS® depression, *T*-score	47 (42, 52)	46 (43, 49)	61 (55, 65)	<0.001

Quantitative variables: median (p25, p75); Categorical variables: *N* (frequency, %).

AF, atrial fibrillation; AFEQT, atrial fibrillation effect on QualiTy-of-life; BMI, body mass index; eGFR, estimated glomerular filtration rate; LA, left atrium; LV, left ventricle; LVEDD, left ventricular end-diastolic diameter; PROMIS^®^, patient-reported outcomes measurement information system; RWT, relative wall thickness; sPAP, systolic pulmonary artery pressure.

### Association of phenogroups with clinical outcomes

At 12 months, the younger cluster exhibited the highest AFEQT scores (88.3 ± 2.2), while the emotive cluster had the lowest (62.0 ± 2.8; *P* < 0.001; *Table 4*; Supplementary material online, *Figure S6*). Although absolute score changes among clusters were not significantly different, the younger group showed the largest reduction in residual impairment (71.6 ± 5.1%; *P* < 0.001) compared with the older (59.2 ± 3.8%) and emotive clusters (36.0 ± 5.0%).

**Table 4:** Comparison of AFEQT scores and changes across patient clusters

	Younger cluster/lone AF (*N* = 59)	Older atherosclerotic cluster (*N* = 100)	Emotive cluster (*N* = 54)	*P*-value
Mean AFEQT overall summary score				
Baseline	58.9 (3.0)	58.4 (2.3)	40.7 (4.0)	<0.001
Month 12	88.3 (2.2)	83.0 (1.7)	62.0 (2.8)	<0.001
Absolute difference	+29.4 (3.1)	+24.7 (2.2)	+21.3 (3.9)	0.241
Relative difference (%)	+50.0 (7.4)	+42.3 (5.1)	+52.4 (13.8)	0.609
Proportion of maximal possible improvement (%)	71.6 (5.1)	59.2 (3.8)	36.0 (5.0)	<0.001
Mean AFEQT subdomains score				
Daily activities				
Baseline	59.2 (3.6)	59.6 (2.7)	36.3 (4.6)	<0.001
Month 12	88.5 (2.6)	82.9 (2.0)	60.2 (3.4)	<0.001
Absolute difference	+29.4 (3.9)	+23.2 (2.6)	+23.9 (4.1)	0.411
Symptoms				
Baseline	66.6 (3.0)	64.8 (2.4)	49.3 (4.7)	0.005
Month 12	89.4 (2.2)	85.7 (1.7)	66.8 (3.1)	<0.001
Absolute difference	+22.8 (3.2)	+20.9 (2.5)	+17.5 (4.7)	0.652
Treatment concerns				
Baseline	53.1 (3.1)	54.7 (2.4)	39.6 (4.4)	0.009
Month 12	87.5 (2.4)	82.2 (1.8)	61.0 (2.9)	<0.001
Absolute difference	+34.5 (3.2)	+27.6 (2.3)	+21.4 (4.3)	0.041

Scores are presented as mean (standard error).

AF, atrial fibrillation; AFEQT, atrial fibrillation effect on QualiTy-of-life.

**Figure 6:**
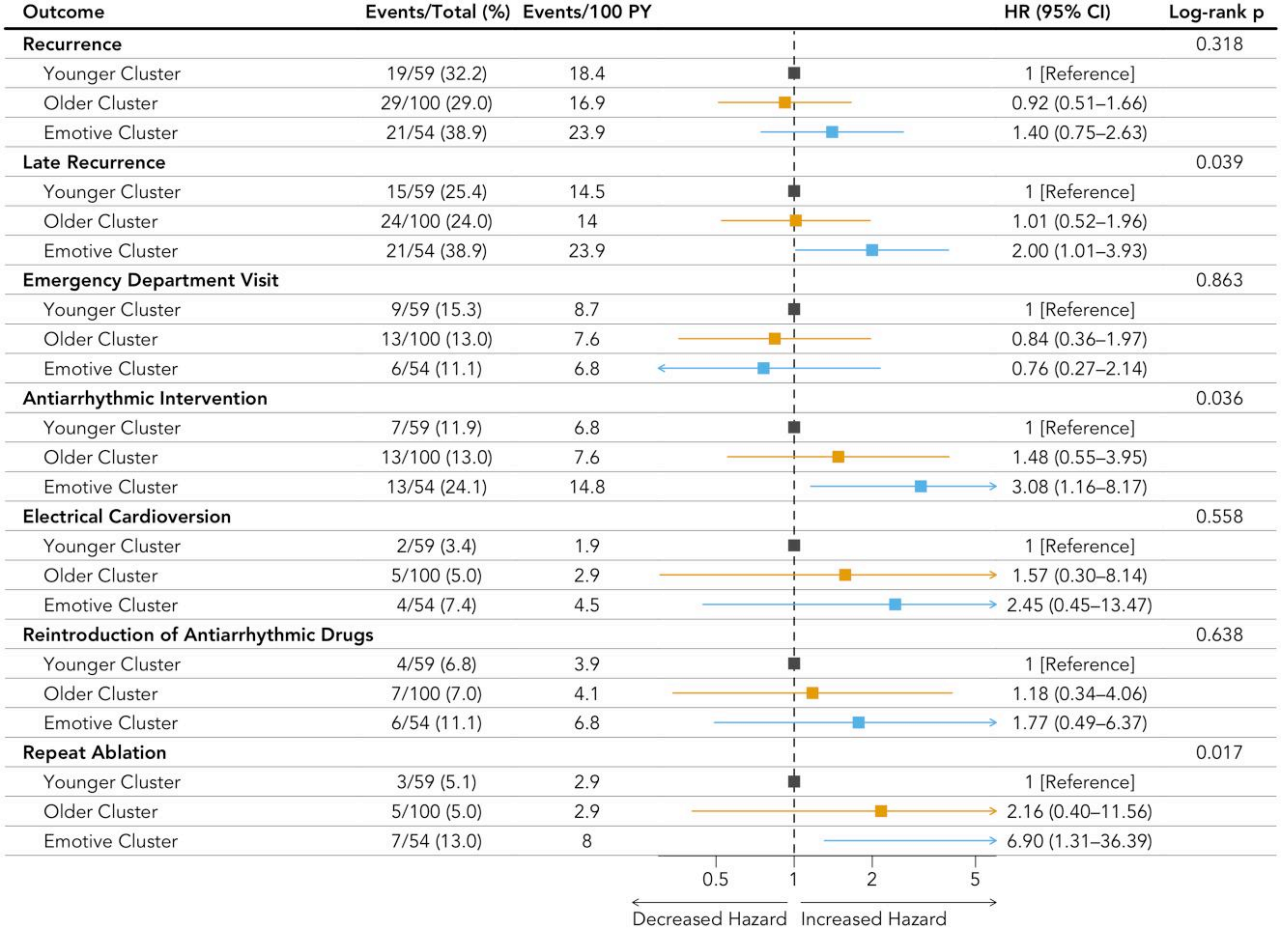
Clinical outcomes stratified by phenogroups. CI, confidence interval; HR, hazard ratio; PY, patients-year.

While the overall rates of any and total arrhythmia recurrence did not differ significantly across clusters (*Figure 6*; Supplementary material online, *Figure S7*), the emotive cluster showed a significantly higher rate of late recurrence [hazard ratio (HR): 2.00, CI: 1.01–3.93; *P* = 0.039] when compared with the reference younger cluster. Furthermore, the emotive cluster also faced a significantly higher rate of any subsequent antiarrhythmic intervention (HR: 3.08, CI: 1.16–8.17; *P* = 0.036) and, specifically, of repeat ablation (HR: 6.90, CI: 1.31–36.39; *P* = 0.017). In contrast, the older cluster did not exhibit a significantly different hazard for late recurrence (HR: 1.01, CI: 0.52–1.96), antiarrhythmic intervention (HR: 1.48, CI: 0.55–3.95), or repeat ablation (HR: 2.16, CI: 0.40–11.56) compared with the younger cluster. No statistically significant differences were observed among the three clusters for the rates of emergency department visits (*P* = 0.863), electrical cardioversion (*P* = 0.558), or the reintroduction of antiarrhythmic drugs (*P* = 0.638). Two MACEs occurred in the emotive cluster, one of which was a post-ablation complication.

## Discussion

By leveraging ePROs collection through a digital app and an ML clustering framework, we identified three clinically coherent phenotypes of AF patients with distinct QoL trajectories. Using readily available clinical variables, our model was able to assign new patients to these phenotypes with high accuracy. Explainable artificial intelligence (AI) analyses revealed how biological and psychosocial factors jointly shape post-ablation QoL, underscoring the need for multidisciplinary care strategies that extend beyond rhythm control.

### Patient-reported outcomes

Rhythm control in patients with AF has evolved from physician-centred paradigms prioritizing mortality (with limited proven benefit) and procedural outcomes towards a patient-focused approach emphasizing symptoms and QoL improvement. Current guidelines highlight the importance of incorporating QoL as a crucial outcome measure in clinical practice and research to assess the efficacy of AF treatments. Recent advancements in digital healthcare have enabled real-time collection of electronic versions of previously paper-based questionnaires, which can be used more frequently and triggered by patients as well as by healthcare professionals, supporting the transition to multidisciplinary integrated care. Using a smartphone mobile application, this study achieved an AFEQT completion rate (92.5% at 6 months, 77% at 1 year) that not only exceed traditional methods (e.g. 75% with emailed QoL questionnaires^16^) but also rival those reported in large RCTs like CABANA (91%)^17^ and STAR AF II (85%).^18^ These results show the potential of digital health to overcome common data-collection barriers like survey fatigue and accessibility limitations, providing an effective and comfortable method for real-time QoL monitoring.

### Predicting quality of life outcomes

Our cohort demonstrated rapid, significant and sustained QoL improvements after AF ablation, consistent with prior RCTs.^17,19,20^ Despite the lack of a sham control in our study, no clinically relevant placebo effect from AF ablation was recently demonstrated in SHAM-PVI trial.^19^ Although the magnitude of QoL improvement at the population level is partially dictated by baseline levels and procedural outcome, there is considerable variability in individual QoL trajectories. This variability likely reflects the heterogeneity of patients’ clinical profiles. Since conditions such as hypertension often co-occur with other comorbidities, outcome disparities are likely to arise from multiple interacting factors rather than any single variable. Traditional regression models have difficulty capturing such complex relationships, particularly in the presence of correlated predictors.^8^ Although unsupervised clustering can group patients who share similar correlated characteristics, it often disregards key outcome data, yielding clusters with limited prognostic relevance (as seen in ORBITA-AF and KiCS-AF^9,10^). On the other hand, clustering techniques using latent class analysis have been used to unveil different outcomes trajectories among AF patients, but lacked integration of clinical characteristics.^21^ Our study builds on previous research by linking baseline characteristics to outcomes data through cluster membership. Using profile regression, we ensured that the identified clusters are both clinically coherent and distinct in their post-ablation QoL trajectories, providing relevant stratification information.^11^ Cross-validation showed that the model could assign patients to one of the three phenogroups with high accuracy using widely available baseline features. Key predictors of these phenotypes include anxiety, age, sex, AF duration, cardiovascular risk factors, and echocardiographic evidence of LV remodelling and pulmonary hypertension. Notably, AF temporal patterns and LAVI proved less discriminative. AF temporal patterns may be inaccurately classified due to the reliance on subjective symptom reports.^22^ Furthermore, a previous study found no evidence that these patterns impacted AF-related QoL.^23^ On the other hand, LAVI may only partially reflect the cumulative influence of multiple factors already captured by the model.

Next, our SHAP analysis revealed that the impact of any single predictor depends on its interactions with other variables, challenging the assumption that a single factor affects all individuals uniformly. For example, anxiety alone does not reliably predict a patient's phenotype or ablation outcome. A patient with high anxiety may experience poor outcomes if their broader clinical profile aligns with the ‘emotive’ cluster, yet they may fare well if they instead resemble the ‘younger’ cluster. These findings underscore that patient trajectories emerge from the interplay of psychological and biological factors, collectively defining a distinct clinical signature.

### Clinical implications

The younger cluster comprised patients with few comorbidities who presented soon after their first paroxysmal AF episode and subsequently experienced a prolonged asymptomatic period. A large proportion of these patients remain free of further recurrence,^24^ with only a minority (7.1 per 100 patient-years) progressing to persistent AF.^25^ While younger patients may be more prone to medication non-adherence and more susceptible to the impact of AF on physical activity, the effects of ablation on treatment concerns and daily activities remain unclear.^26^ In addition, evidence regarding ablation success in this population is mixed.^27,28^ However, our findings suggest that AF ablation is a viable option for rhythm control and QoL beyond mere symptom relief. Notably, time since diagnosis did not limit QoL benefits in this young group, likely because of their lower AF burden and minimal LA remodelling. Although a longer interval to ablation has been linked to higher recurrence rates,^29^ recent randomized trials indicate that delaying ablation while adopting a conservative approach does not compromise long-term ablation outcomes.^30^

The older cluster resembled the atherosclerotic-comorbid cluster in ORBIT-AF.^10^ Although these patients had a higher prevalence of cardiovascular risk factors, they experienced no MACE and demonstrated ablation outcomes comparable to the less comorbid cluster. Despite limited FUP, these findings may reflect healthier lifestyle changes and stricter control of modifiable risk factors, as patients often adopt better behaviours when enrolled in monitoring programmes.^31^ This hypothesis is consistent with recent findings from a sub-analysis of TeleCheck-AF project, where older age was an independent predictor of adherence and motivation to on-demand heart rate/rhythm monitoring app.^32^

The emotive cluster comprised patients with the highest anxiety levels and the lowest AFEQT scores, consistent with evidence suggesting a bidirectional relationship in which AF triggers anxiety and depression, which in turn amplify AF perception.^33^ This cluster also exhibited a significantly higher recurrence rate than other clusters, aligning with prior findings linking anxiety to increased AF recurrence after ablation.^34^ Concurrently, the distinctive echocardiographic pattern (elevated sPAP without LV remodelling) uncovered by SHAP analysis, together with larger LA volumes, suggests that these patients may have more advanced atrial remodelling or diastolic dysfunction that would provide a substrate for AF perpetuation. Whether anxiety is a marker, a by-product, or a promoter of cardiovascular multimorbidity remains elusive.^35–37^ Notably, overall QoL in this cluster improved less than in others, even with increased antiarrhythmic reinterventions. While these patients experienced symptomatic and functional relief, they showed less improvement in the treatment concerns subdomain compared with other clusters, suggesting that underlying anxiety about their condition and its ongoing management may persist. Antiarrhythmic reinterventions often included resuming beta-blockers (BB) and antiarrhythmic drugs (AAD), which can induce neuropsychiatric side effects, thereby limiting QoL.^38,39^ This hypothesis is supported by a recent RCT, which, in addition to demonstrating ablation's superiority over medical therapy in reducing psychological distress and AF symptoms, also showed that discontinuing BB/AAD improved psychological well-being.^40^ Further research is warranted to clarify the risk-benefit ratio of antiarrhythmic therapy in patients with significant anxiety or depression, ensuring that neither sinus rhythm nor QoL is compromised. Cognitive behavioural therapy has recently been proven to enhance health-related QoL and reduce psychological distress in AF patients.^41^ Similarly, psychotherapy and antidepressant drugs have been linked to decreased hospitalizations and mortality in patients with heart disease.^42^ Studies are needed to determine whether mental health interventions may improve QoL in post-ablation patients and lessen the side effects of BB/AAD.^43^ Despite international guidelines acknowledging the psychological morbidity of AF patients, current AF management often overlooks key aspects such as anxiety and depression.^44^ More recently, a multidisciplinary approach (or care model) has been encouraged to optimize patient-centred management,^1^ and the integration of psychologists into the AF care team has been advocated.^44^

By clarifying how psychological and biological traits interact to shape QoL, our framework can help identify patients who may be more susceptible to the neuropsychiatric effects of BB/AAD or likely to benefit from psychological interventions. Phenotype-based profiling has the potential to facilitate tailored multidisciplinary care that extends beyond individualized rhythm control strategies.

## Limitations

Our study's single-centre origin, limited sample size, and distinct demographics (largely Caucasian, with fewer women and patients with persistent AF) restrain the broad applicability of our results. Given that the ML-identified clusters probably reflect the dominant phenotypes specific to our cohort, further investigation in larger, more varied populations is essential to validate these clusters’ stability and relevance, and to identify other potential patient subgroups. Despite robust imputation techniques and sensitivity analyses, the initial extent of missing data remains a constraint. This was particularly evident for the baseline AFEQT questionnaire, with an approximate 50% completion rate stemming from initial ePRO system implementation challenges. The absence of a sham-controlled group introduces the possibility of a placebo effect. However, the sustained 12-month correlation between QoL improvements and ablation efficacy, coupled with recent SHAM-PVI trial findings, suggests any such influence was likely minimal.^19^ The requirement for digital app registration might have introduced selection bias by potentially excluding less digitally literate individuals. Finally, the absence of continuous implantable monitors could have under-detected AF events, although frequent transtelephonic monitoring during follow-up helped capture them.^2^

## Conclusion

This prospective study highlights the value of integrating ePROs with advanced analytics to find coherent clinical profiles with prognostic relevance among heterogeneous clinical population undergoing first-time AF ablation. Using explainable AI, we further clarified how biological and psychosocial factors interact to shape post-ablation trajectories, thereby facilitating personalized multidisciplinary management strategies beyond rhythm control that may integrate psychological support.

## Supplementary Material

ztaf090_Supplementary_Data

## Data Availability

The data underlying this article will be shared on reasonable request to the corresponding author.
