# Supplementary material for: Predicting patient-related outcomes after atrial fibrillation ablation: insights from explainable artificial intelligence and digital health
Source: Eur Heart J Digit Health. 2025 Aug 7;6(6):1181–93. doi: 10.1093/ehjdh/ztaf090 (PMC12629652; doi:10.1093/ehjdh/ztaf090)
Supplement: ztaf090_Supplementary_Data [file ztaf090_supplementary_data.docx]

**SUPPLEMENTARY MATERIAL**

**SUPPLEMENTARY METHODS**

## **Catheter ablation procedures**

## Catheter ablation procedures included pulmonary vein isolation (PVI) with point-by-point radiofrequency and second-generation cryoablation at the discretion of the operator. All procedures were performed under sedation and with continuous hemodynamic and electrocardiographic monitoring. Femoral venous access was used in all procedures. Transseptal puncture was performed with fluoroscopic guidance. The procedural endpoint was the electrical isolation of all pulmonary veins. Cavotricuspid isthmus ablation was performed concomitantly in selected patients with atrial flutter, and ablation of additional lesions was performed at the operator's discretion.

**Analysis of longitudinal AFEQT data missingness mechanisms**

A two-step sensitivity analysis was performed to evaluate the impact of departures from the Missing-At-Random (MAR) assumption in our longitudinal AFEQT data.  First, we used Multiple Imputation by Chained Equations (MICE) with a patient-level random intercept (20 imputations) to generate complete data sets under MAR.  In each imputed set we calculated the 12-month change in AFEQT score and then pooled the mean change, and variance across imputations via Rubin’s rules. After imputation, we assessed the impact of potential violations of the MAR assumption through pattern-mixture model sensitivity analyses. These analyses systematically decreased/increased imputed AFEQT values by specified amounts (Δ = –20, –15, –10, –5, 0, +5, +10, +15 and +20 points), effectively assuming missing values were progressively worse/better than predicted under MAR conditions. For each delta-adjusted dataset, we recalculated AFEQT changes from baseline to 12 months and again pooled by Rubin’s rules. This pattern-mixture approach quantifies how estimated treatment effects would differ if every imputed score were too low or too high by Δ, approximating a worst-case/ best-case Missing-Not-At-Random (MNAR) scenario.

**Longitudinal changes in AF related QoL- linear mixed-effects model (LMM)**

The optimal number and location of knots for the splines were determined by the lowest mean squared error in 5-fold cross-validation (CV). Restricted maximum likelihood estimation with an unstructured covariance matrix was used to model all available scores. No explicit imputation was needed for population estimates as LMMs handle incomplete data and varying follow-up lengths.

**Association between patient-reported outcomes and clinical outcomes**

The association between patient-reported outcomes and clinical outcomes (AF recurrence) was tested through the development of normative reference curves derived from the non-recurrence cohort. In the first stage, referred to as the curve smoothing stage, selected empirical percentiles were smoothed using penalized B-splines within the Generalized Additive Models for Location, Scale, and Shape (GAMLSS) framework. This nonparametric regression technique flexibly models the relationship between time and AFEQT scores while mitigating overfitting risk. In the second stage, known as the transformation stage, we employed modified Lambda-Mu-Sigma (LMS) parameterization (λ, μ, σ) to convert bounded AFEQT scores into standardized Z-score distributions, effectively addressing ceiling effects and heteroscedasticity across temporal assessment points. These Z-scores quantified how individual patients’ QoL deviated from the cohort average at specific post-ablation time points. This enabled the construction of final percentile curves that closely matched the initial smoothed curves and the computation of additional percentiles and Z-scores. Subsequently, a Linear Mixed Model (LMM) with random intercepts was used to analyse the interaction between time and relapse status on the AFEQT Z-scores. This analytical structure enabled robust isolation of recurrence-attributable effects through conditional standardization against reference populations, addressing non-linear temporal variance structures and baseline heterogeneity.

**Machine Learning**

1. **Data preprocessing**
   1. **Multiple Imputation**

Multilevel joint modeling with multiple imputation integrates information across related data points, preserving the inherent structure of the dataset and improving the plausibility and precision of imputed values. The method operates by defining a joint distribution for all variables in the model, combining the distributions of baseline covariates with the substantive model used for the outcome of interest (i.e., conditional model for the longitudinal outcome given the covariates). The joint imputation model was implemented using a multivariate normal model via the *jomo* R package, which employs a Markov Chain Monte Carlo (MCMC) algorithm to generate imputations iteratively. Auxiliary covariates used in the joint imputation model (detailed in **Supplementary Table 2)** included demographic data (age, sex), clinical variables (body mass index [BMI], diabetes mellitus, hypertension, dyslipidemia, history of smoking, history of stroke or transient ischemic attack, coronary artery disease, heart failure), biomarkers (creatinine, hemoglobin), psychometric data (PROMIS® anxiety and depression score), AF variables (type of AF, duration since first diagnosis of AF, heart rate), echocardiographic variables (relative wall thickness, early diastolic mitral annulus e’ tissue velocity, mitral E-wave velocity, mitral velocity deceleration time, mitral velocity A-wave, indexed left ventricular [LV] volume, indexed LV mass, LV ejection fraction, pulmonary systolic arterial pressure, tricuspid annular plane systolic excursion, right atrial area, left atrial volume index, pulmonary vein acceleration-to-ejection time ratio, moderate-to severe mitral and tricuspid regurgitation [≥ III/IV color Doppler grade]) and areas of fibrosis in electroanatomic mapping. To represent the maximum uncertainty and to give the greatest weight to the data, we used flat improper priors for all the parameters in our joint imputation model, except the cluster-specific covariance matrices, for which random covariance structures were adopted to allow for heteroskedasticity. The model ran 1,000 burn-in iterations.

- 1. **Principal Component Analysis (PCA)**

After imputing missing data, PCA was performed on echocardiographic variables as a means of dimensionality reduction. This step aimed to reduce the number of input variables to make the subsequent clustering process more robust and to identify common patterns of structural and functional variation within the dataset. PCA derives a new set of variables, known as principal components, through linear combinations of the original features. These components are ordered based on the amount of variance they explain in the dataset, with the first principal component capturing the greatest variability, followed by subsequent components that account for progressively smaller proportions of the total variance. PCA was conducted separately for each imputed dataset to account for variability introduced by imputation. Based on the number of components with eigenvalues > 1 and variance explained > 50%, a consistent number of principal components was selected using a majority vote across all imputed datasets^1^.

1. **Profile regression Mixture Models (Semisupervised learning)**
   1. **Clustering model**

Our hypothesis suggested that, regardless of the diverse characteristics of AF patients, certain phenotypic elements would emerge in consistent patterns, indicating the predominant influence of one or more underlying pathological factors. To identify these recurring patterns, we utilized profile regression mixture modeling, a semisupervised machine learning technique. This method combines unsupervised clustering to reveal inherent data structures with an outcome-focused approach that prioritizes patient groupings linked to specific outcomes. We implemented this analysis using the *PReMiuM* R package, which simultaneously develops two models: an "assignment model" for categorizing patients into clusters and a "disease submodel" that connects these clusters to an outcome through regression. The assignment model employs a Dirichlet process prior for the mixing distribution, allowing the number of clusters to be automatically determined during the model fitting process.

Inputs variables included previously defined demographic, clinical, psychometric and AF variables, as well as the main echocardiography PCA principal components. Before clustering, continuous variables were normalized, and discrete variables were transformed into factors. Through MCMC sampling, the algorithm iteratively updated both models. Each MCMC iteration generates a unique patient clustering, and an optimal grouping is achieved by applying a partitioning algorithm (partitioning around medoids) across all sampled iterations. The model was fit separately in each imputed datasets, with 40,000 burn-in and 40,000 sampling iterations. Clustering results for each imputed dataset were then pooled to generate a consensus cluster.

- 1. **Consensus clustering**

Clustering results from multiple imputed datasets were aggregated into a co-occurrence matrix, which was normalized and used for consensus clustering with the *ConsensusClusterPlus* R package to identify stable clusters. The consensus clustering process was performed on 1,000 bootstrap samples of 80% of data points in each iteration, using all features in every iteration, employing hierarchical clustering and using Pearson correlation distance. To identify the ideal number of clusters (k), we selected the k value that showed the highest agreement across the imputed datasets. We then verified the clustering outcomes by computing silhouette scores, which measure how well objects fit within their assigned clusters and how distinct they are from other clusters. Additionally, we utilized the elbow technique to pinpoint the optimal cluster count by graphing the within-cluster sum of squares (WSS). Additionally, the Calinski-Harabasz and Davies-Bouldin indices were computed using the *clusterCrit* R package to quantitatively measure clustering quality. Final clusters were assigned to the original data to form phenogroups.

1. **Identifying Key Predictors of Patient Phenogroups (Supervised learning)**
   1. **XGBOOST (Extreme Gradient Boosting)**

Once clusters were defined, a classification model was trained using the clustered dataset. With this, the model learns which features are most important for differentiating clusters. In practice, this transforms the initially discovered patterns into a predictive tool that can classify new data into the previously found groups. This two-step approach ensures the clusters are not statistical artifacts but represent distinct, clinically relevant groups characterized by identifiable predictors. XGBoost is an advanced machine learning algorithm that improves predictions by combining the strengths of many smaller, simpler models called decision trees. It works iteratively: each new tree is built to correct the errors made by the previous ones, gradually refining the model’s accuracy. The algorithm incorporates regularization techniques to prevent overfitting, ensuring that the model generalizes well to new data.

To mitigate the risk of model overfitting to biases or excessively specific patterns within the training data, which can happen in small cohorts, we implemented feature selection and cross-validation techniques.

- 1. **Feature selection**

To enhance model accuracy and improve interpretability, we implemented a structured feature selection process. First, we filtered out highly correlated variables (Pearson correlation >0.7) by removing those with the highest mean absolute correlation, thereby reducing redundancy. Next, we applied simulated annealing to minimize cross-entropy loss and refine the feature set. Finally, an ensemble method using majority voting from models trained on multiple imputed datasets ensured the selection of consistently important predictors. This systematic approach produced a more parsimonious feature set that maintained predictive performance while increasing transparency and robustness.

- 1. **Model Tunning and evaluation**

We employed a nested 5-fold cross-validation with stratification at both levels for model training and validation. This method involves two levels of data splitting.

In the inner loop, hyperparameter tuning via grid search was conducted, where different hyperparameter combinations were evaluated through cross-validation to find the best performing set. The final model was configured with 500 boosting rounds to ensure sufficient training iterations for convergence. A maximum tree depth of 3 was chosen to control model complexity and prevent overfitting. The learning rate was set to 0.007, balancing the trade-off between training speed and accuracy. The Lagrange multiplier was 0, and the L2 regularization weight was 1. Half of the features were randomly sampled for each tree. The minimum number of points per node was set to 2, ensuring robustness against noisy data. Lastly, we utilized a subsample ratio of 1, meaning that all training data was used for each boosting round, which helps in stabilizing the model.

The outer loop provides an unbiased evaluation of the model's performance using a separate data split after hyperparameter tuning, ensuring the model's generalizability. Because the hyperparameter tuning step includes its own cross-validation, this results in a nested cross-validation structure.

Model performance metrics on each imputed dataset were then estimated based on this comprehensive approach and combined using Rubin’s Rules.

**Endpoints**

Repeat ablations were generally delayed until 6 months after the initial ablation procedure.

**Recurrent event analyses**

We analyzed recurrent event data using a negative binomial regression model, incorporating the logarithm of each individual's follow-up time (in days) as an offset term to account for varying exposure. Incidence rate ratios (RRs) with 95% confidence intervals were estimated from this model to compare event rates across clusters, and the overall statistical significance of the cluster variable was assessed using a robust Wald test.

**SUPPLEMENTARY RESULTS:**

**Analysis of longitudinal AFEQT data missingness mechanisms**

Under the MAR assumption, the pooled mean AFEQT improvement from baseline to 12 months was +23.2 ± 2.0 points (95 % CI 19.3–27.1). Applying shifts to all imputed values changed the mean AFEQT improvement estimate by less than 5 points relative to MAR (**Supplementary Table 5**).  Even in the most extreme scenario, the treatment effect remained well above any minimal clinically important difference.

**Recurrent event analysis**

Overall, there was no statistically significant difference in total recurrence rates among the patient clusters (P = 0.112), although the Emotive Cluster showed a tendency towards a higher, non-significant recurrence rate (RR: 1.71, CI: 0.92–3.23) compared to the Younger Cluster (**Supplementary Figure 7**).

**SUPPLEMENTARY TABLES**

**Supplementary Table 1-** Follow-up program

| **Evaluation** | **Pre-Ablation** | **Procedure** | **1 Month** | **3 Months** | **6 Months** | **9 Months** | **12 Months** |
| --- | --- | --- | --- | --- | --- | --- | --- |
| **In-Person Consultation (clinical evaluation)** | **X** | **X** |  |  |  |  | **X** |
| **Telephone Consultation (symptom evaluation)** |  |  | **X** | **X** | **X** |  |  |
| **Self-Recording via Promptly® App** |  |  | **X** | **X** | **X** | **X** | **X** |
| **Electrocardiogram** | **X** | **X** | **X** | **X** | **X** |  | **X** |
| **Holter (24h)** | **X** |  |  |  |  | **X** |  |
| **Transthoracic Echocardiogram** | **X** | **X** |  |  |  |  |  |
| **Cardiac CT Scan** | **X** |  |  |  |  |  |  |
| **Questionnaires (about symptoms and QoL)** | **X** |  | **X** | **X** | **X** |  | **X** |

QoL: Quality of Life

**Supplementary Table 2**- Missingness of variables used in clustering model

| **Variables** | **Missingness (%)** |
| --- | --- |
| **Demographic** |  |
| Age | 0 |
| Sex | 0 |
| **Clinical** |  |
| Body mass index | 0 |
| Diabetes mellitus | 0 |
| Hypertension | 0 |
| Dyslipidemia | 0 |
| History of smoking | 0 |
| History of stroke or transient ischemic attack | 0 |
| Coronary artery disease | 0 |
| Heart failure | 0 |
| **Biomarkers** |  |
| Creatinine | 5.1 |
| Hemoglobin | 4.7 |
| **Psychometric** |  |
| PROMIS® anxiety score | 47.1 |
| PROMIS® anxiety score | 47.1 |
| **AF** |  |
| Type of AF | 0 |
| Duration since first diagnosis of AF | 0 |
| Heart rate | 11.4 |
| **Echocardiographic*** |  |
| Relative wall thickness | 7.2 |
| Mitral annulus e’ tissue velocity | 39.4 |
| Mitral E-wave velocity | 9.7 |
| Mitral velocity deceleration time | 10.6 |
| Mitral velocity A-wave | 11.4 |
| indexed LV volume | 9.3 |
| indexed LV mass | 6.4 |
| LV ejection fraction | 3.4 |
| PSAP | 27.5 |
| TAPSE | 10.2 |
| Right atrial area | 11.4 |
| Left atrial volume index | 5.5 |
| Pulmonary vein acceleration-to-ejection time ratio | 13.6 |
| **Other** |  |
| Areas of fibrosis | 22.5 |

*Principal Component Analysis was performed on echocardiographic variables before entering the model.

AF: Atrial Fibrillation; BMI: Body Mass Index; LV: Left Ventricular; MR: Mitral Regurgitation; PSAP: Pulmonary Systolic Arterial Pressure; TAPSE: Tricuspid Annular Plane Systolic Excursion; TR: Tricuspid Regurgitation.

**Supplementary Table 3-** Patterns of missing data

| **Pattern** | **T= 0** | **T= 1 month** | **T= 3 months** | **T= 6 months** | **T= 12 months** | **Number of patients** | **Relative frequency (%)** |
| --- | --- | --- | --- | --- | --- | --- | --- |
| **AAAAA** | 55 | 72 | 79 | 79 | 76 | 44 | 20,7 |
| **MAAAA** |  | 73 | 79 | 81 | 78 | 42 | 19,7 |
| **AMAAA** | 56 |  | 74 | 78 | 76 | 18 | 8,5 |
| **MMAAA** |  |  | 76 | 81 | 75 | 14 | 6,6 |
| **MAAAM** |  | 66 | 71 | 79 |  | 13 | 6,1 |
| **AAAAM** | 44 | 72 | 68 | 76 |  | 10 | 4,7 |
| **AMMAA** | 52 |  |  | 80 | 81 | 7 | 3,3 |
| **MAAMM** |  | 77 | 77 |  |  | 7 | 3,3 |
| **MMMAA** |  |  |  | 71 | 73 | 6 | 2,8 |
| **AMAMA** | 54 |  | 82 |  | 82 | 5 | 2,3 |
| **MAMAA** |  | 47 |  | 68 | 71 | 4 | 1,9 |
| **MAMMM** |  | 63 |  |  |  | 4 | 1,9 |
| **MMAAM** |  |  | 70 | 70 |  | 4 | 1,9 |
| **MMAMA** |  |  | 60 |  | 81 | 4 | 1,9 |
| **MMMMA** |  |  |  |  | 64 | 4 | 1,9 |
| **AAAMA** | 57 | 67 | 82 |  | 78 | 3 | 1,4 |
| **AAAMM** | 51 | 94 | 92 |  |  | 3 | 1,4 |
| **AAMAM** | 43 | 69 |  | 67 |  | 3 | 1,4 |
| **MMMAM** |  |  |  | 73 |  | 3 | 1,4 |
| **AAMAA** | 61 | 44 |  | 76 | 87 | 2 | 0,9 |
| **AAMMA** | 39 | 44 |  |  | 56 | 2 | 0,9 |
| **AAMMM** | 83 | 70 |  |  |  | 2 | 0,9 |
| **AMMMA** | 78 |  |  |  | 76 | 2 | 0,9 |
| **AMMMM** | 64 |  |  |  |  | 2 | 0,9 |
| **MAMAM** |  | 49 |  | 75 |  | 2 | 0,9 |
| **AMAAM** | 98 |  | 93 | 100 |  | 1 | 0,5 |
| **AMAMM** | 48 |  | 49 |  |  | 1 | 0,5 |
| **MAMMA** |  | 68 |  |  | 25 | 1 | 0,5 |

Comprehensive characterization of data completeness across five assessment timepoints (baseline, 1, 3, 6, and 12 months). Pattern codes indicate availability status at each successive timepoint T (A=available, M=missing). Distribution presented as absolute frequency and relative percentage of the total cohort (N=213). Modal pattern was complete data ascertainment (AAAAA: 20.7%), followed by baseline-only omission (MAAAA: 19.7%). Cell values within patterns represent mean AFEQT scores for available timepoints.

**Supplementary Table 4-** Baseline characteristics according to responder status

|  | **Total**  **(n= 236)** | **Responder**  **(n= 213)** | | **Non-responder**  **(n= 23)** | **P-value** |
| --- | --- | --- | --- | --- | --- |
| **Demographics** | | | | | |
| **Age (years)** | 60 (51, 67) | 60 (51, 67) | | 62 (58, 67) | 0.379 |
| **Female** | 75 (32) | 65 (31) | | 10 (43) | 0.302 |
| **BMI (kg/m^2^)** | 27.3 (24.8, 29.4) | 27.1 (24.8, 29.4) | | 28.5 (25.9, 29.1) | 0.464 |
| **FUP (months)** | 25.4 (18.9, 31.7) | 26.1 (19.3, 31.9) | | 21.9 (16.4, 27.1) | 0.060 |
| **Arrhythmia** | | | | | |
| **Atrial fibrillation** |  |  | |  | 0.180 |
| **Paroxysmal** | 189 (80) | 173 (81) | | 16 (70) |  |
| **Persistent** | 47 (20) | 40 (19) | | 7 (30) |  |
| **CHA_2_DS_2_VASc** |  |  | |  | 0.113 |
| **0-1 (Lowest risk)** | 129 (55) | 129 (57) | | 9 (39) |  |
| **2** | 55 (23) | 50 (23) | | 5 (22) |  |
| **3** | 39 (17) | 33 (15) | | 6 (26) |  |
| **≥ 4 (Highest risk)** | 13 (5) | 10 (5) | | 3 (13) |  |
| **Years since onset of AF** | 2.5 (1.5, 6.5) | 2.5 (1.5, 5.5) | | 3.5 (2.5, 7.5) | 0.252 |
| **No prior direct cardioversion** | 90 (45) | 79 (44) | | 11 (61) | 0.241 |
| **History of Atrial Flutter** | 43 (18) | 38 (18) | | 5 (22) | 0.581 |
| **Prior ablation for atrial flutter** | 4 (2) | 3 (1) | | 1 (4) | 0.338 |
| **Anti-arrhythmic drugs** | 150 (64) | 131 (62) | | 19 (83) | 0.077 |
| **Beta-blockers** | 160 (68) | 147 (69) | | 13 (57) | 0.325 |
| **Procedure** | | | | | |
| **Energy** | |  | |  | 0.054 |
| **Crioenergy** | 49 (21) | 48 (23) | | 1 (4) |  |
| **Radiofrequency** | 187 (79) | 165 (77) | | 22 (96) |  |
| **Cavo-tricuspid isthmus ablation** | 34 (14) | 30 (14) | | 4 (17) | 0.753 |
| **Posterior wall ablation** | 22 (9) | 21 (10) | | 1 (4) | 0.705 |
| **Comorbidities** | | | | | |
| **Thyroid Dysfunction** | 26 (11) | 24 (11) | 2 (9) | | 1 |
| **History of Smoking** | 56 (24) | 49 (23) | 7 (30) | | 0.591 |
| **Dyslipidemia** | 118 (50) | 107 (50) | 11 (48) | | 1 |
| **Heart failure** | 25 (11) | 21 (10) | 4 (17) | | 0.280 |
| **Arterial hypertension** | 115 (49) | 100 (47) | 15 (65) | | 0.148 |
| **Diabetes** | 21 (9) | 19 (9) | 2 (9) | | 1 |
| **Cerebrovascular disease** | 15 (6) | 13 (6) | 2 (9) | | 0.646 |
| **Coronary artery disease** | 11 (5) | 8 (4) | 3 (13) | | 0.080 |
| **Echocardiographic** |  |  |  | |  |
| **LA volume index (mL/m^2^)** | 38 (32, 45) | 37 (31, 45) | 42 (34, 51) | | 0.158 |
| **LV Ejection fraction (%)** | 60 (56, 63) | 60 (56, 63) | 60 (57, 61) | | 0.766 |
| **LV mass index (mg/m^2^)** | 91 (76, 103) | 90 (76, 102) | 93 (78, 103) | | 0.487 |
| **eGFR (mL/min/1.73m^2^)** | 87 (77, 100) | 87 (77, 98) | 87 (71, 106) | | 0.892 |
| **Hemoglobin (g/dL)** | 14.6 (13.5, 15.4) | 14.7 (13.5, 15.4) | 14.1 (13.4, 15.5) | | 0.528 |

Quantitative variables: median (p25, p75); Categorical variables: N (frequency, %). AF: Atrial Fibrillation; BMI: Body Mass Index; eGFR: Estimated Glomerular Filtration Rate; FUP: Follow-up; LA: Left Atrium; LV; Left Ventricle.

**Supplementary Table 5**- Sensitivity of pooled 12-month AFEQT change to shifts in imputed values

| **Δ shift** | **Mean change (12 months − baseline)** | **Difference vs MAR** |
| --- | --- | --- |
| –20 | 28.1 ± 2.2 | 4.9 |
| –15 | 26.9 ± 2.1 | 3.7 |
| –10 | 25.6 ± 2.0 | 2.5 |
| –5 | 24.4 ± 2.0 | 1.2 |
| 0 | 23.2 ± 2.0 | 0 |
| 5 | 22.0 ± 1.9 | –1.2 |
| 10 | 20.8 ± 1.8 | –2.4 |
| 15 | 19.7 ± 1.7 | –3.5 |
| 20 | 18.5 ± 1.7 | –4.7 |

Pooled mean change in AFEQT score at 12 months under MAR assumption and after applying uniform shifts (Δ) to all imputed values only. “Difference vs MAR” indicates the departure from the no-shift (Δ = 0) estimate.

Δ: shift applied to imputed values (points); MAR: missing at random.

**Supplementary Table 6-** XGBoost (One-vs-All) classification performance in 5-fold nested cross-validation across 20 imputed datasets

| **Imputation** | **Mean Weighted F1** | **Mean Accuracy Weighted** | **Mean Precision Weighted** | **Mean AUC** | **Mean Recall Weighted** | **Mean Specificity**  **Weigthed** |
| --- | --- | --- | --- | --- | --- | --- |
| **1** | 83,8 | 84,0 | 85,2 | 94,5 | 82,6 | 89,2 |
| **2** | 82,1 | 82,2 | 84,2 | 93,3 | 79,1 | 88,6 |
| **3** | 84,1 | 84,1 | 84,8 | 94,3 | 83,1 | 90,7 |
| **4** | 82,7 | 82,6 | 84,9 | 95,3 | 82,9 | 89,0 |
| **5** | 81,0 | 81,7 | 82,0 | 93,8 | 80,3 | 88,7 |
| **6** | 85,7 | 85,9 | 86,9 | 95,3 | 84,4 | 91,2 |
| **7** | 85,0 | 84,9 | 85,6 | 94,2 | 83,7 | 90,8 |
| **8** | 80,1 | 80,7 | 81,6 | 93,3 | 79,6 | 87,8 |
| **9** | 83,2 | 83,6 | 84,7 | 95,0 | 82,8 | 89,8 |
| **10** | 81,6 | 81,7 | 83,8 | 94,0 | 81,3 | 88,4 |
| **11** | 81,1 | 81,3 | 81,0 | 95,2 | 79,5 | 89,1 |
| **12** | 84,9 | 85,0 | 86,7 | 95,0 | 84,5 | 90,6 |
| **13** | 81,6 | 81,7 | 82,7 | 93,2 | 80,5 | 88,4 |
| **14** | 79,3 | 79,9 | 81,8 | 93,3 | 77,6 | 86,9 |
| **15** | 84,8 | 84,9 | 86,5 | 95,8 | 83,6 | 90,3 |
| **16** | 82,8 | 83,1 | 85,4 | 94,7 | 83,0 | 89,6 |
| **17** | 82,3 | 82,6 | 85,5 | 95,5 | 80,4 | 88,7 |
| **18** | 82,4 | 82,6 | 84,8 | 95,9 | 81,1 | 89,0 |
| **19** | 84,6 | 84,5 | 87,7 | 95,4 | 82,5 | 90,6 |
| **20** | 81,4 | 81,7 | 82,0 | 94,2 | 78,8 | 88,5 |
| **Mean** | 82,7 | 82,9 | 84,4 | 94,6 | 81,6 | 89,3 |
| **SE** | 6,2 | 6,1 | 7,0 | 3.1 | 6,9 | 3,9 |

CV: cross-validation; SE: standard error; AUC: Area under curve.

**SUPPLEMENTARY FIGURES**

**Supplementary Figure 1-** Timeline of follow-up

|  |
| --- |

Hybrid follow-up program including scheduled visits and remote monitoring appointments. PREM: Patient-Reported Experience Measurements; PROM: Patient-Reported Outcome Measurements.

**Supplementary Figure 2-** Principal component analysis

| 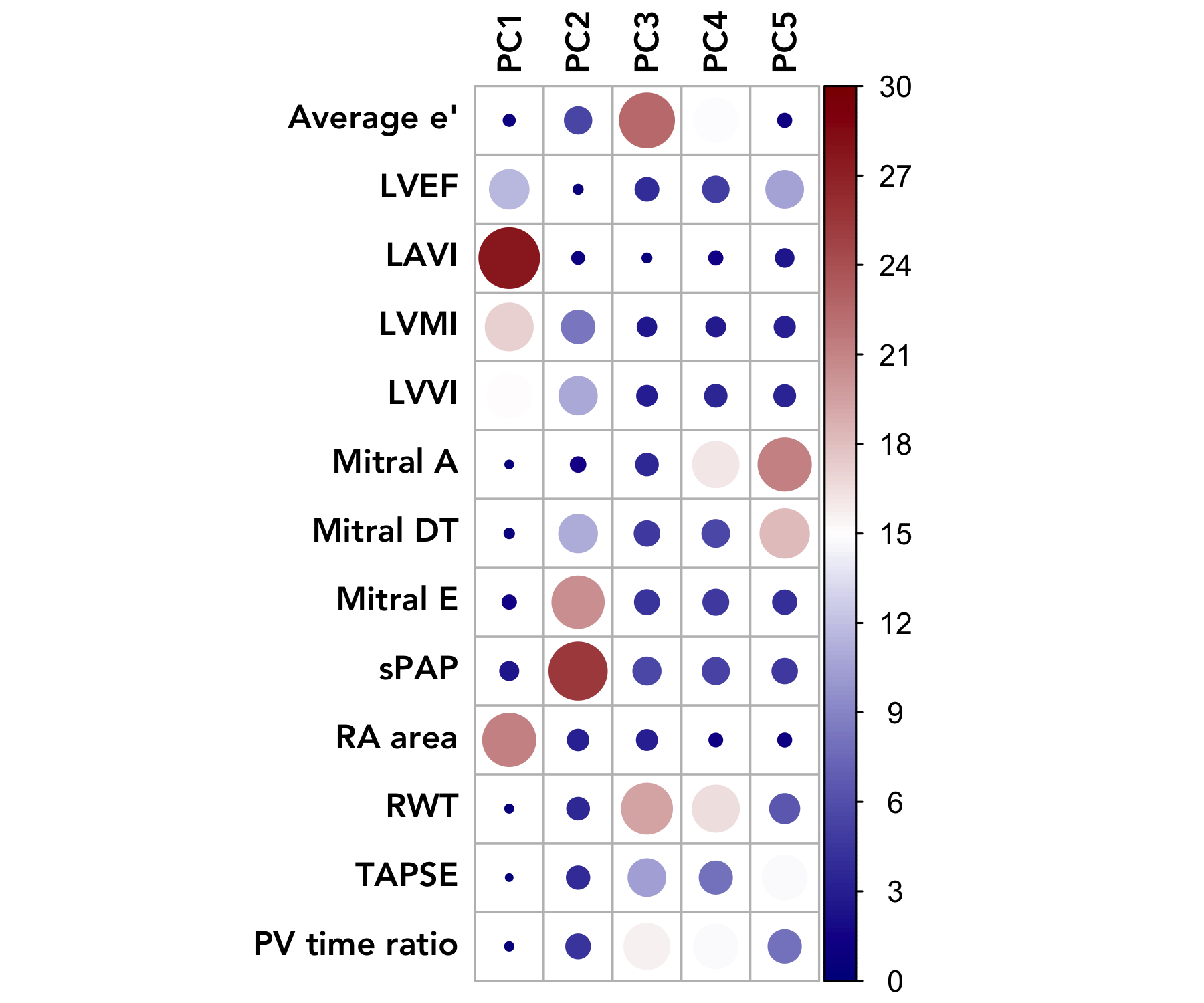 | 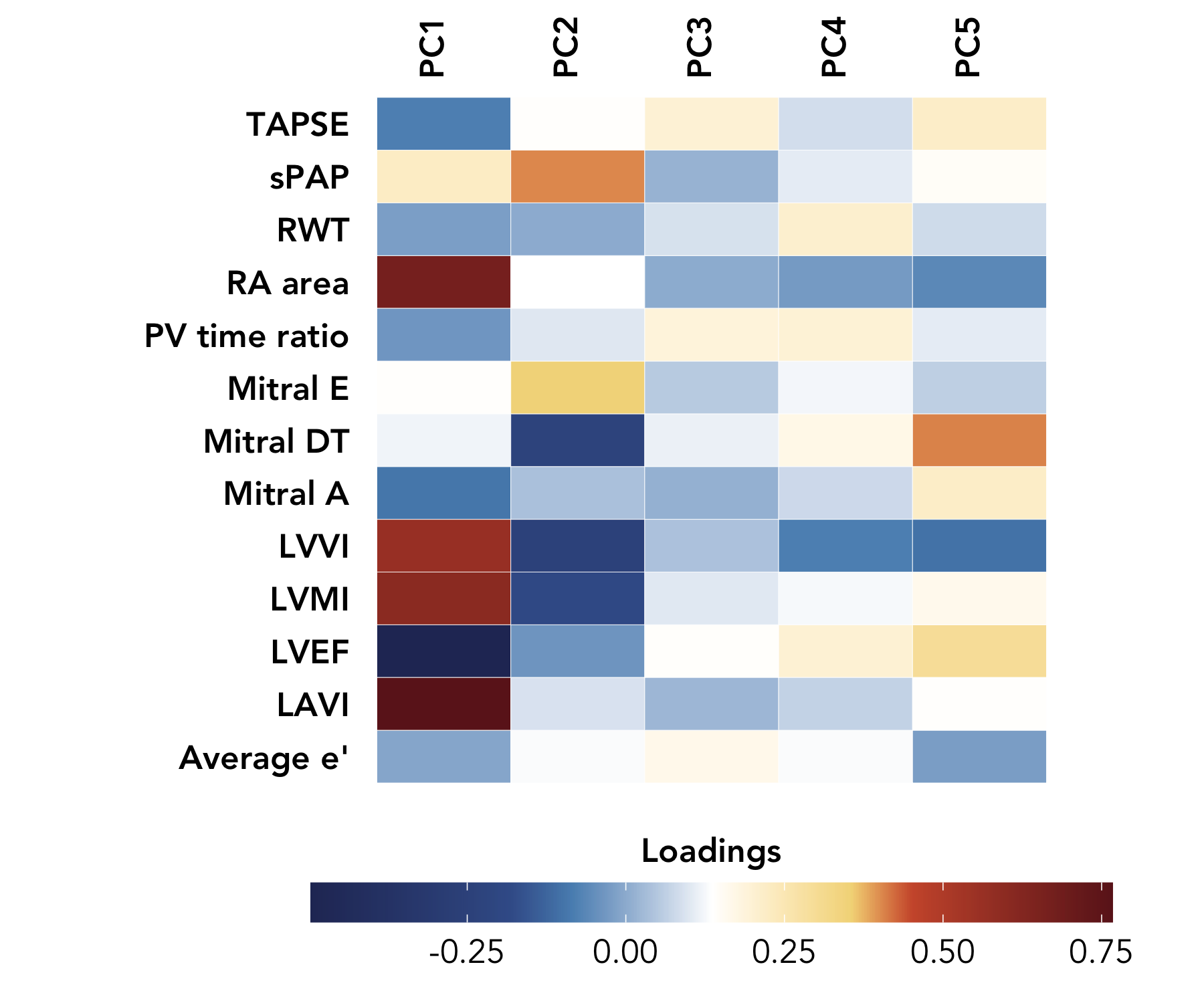 |
| --- | --- |

**(A)** This plot shows the contributions of echocardiographic variables to the first five principal components (PC1 to PC5). Each circle represents the contribution of a variable to a specific component. The color and size of the circles indicate the magnitude of the contribution, with red tones indicating higher contributions and blue tones indicating lower contributions. **(B)** The heatmap displays the loadings of each echocardiographic variable on the first five principal components (PC1 to PC5). The colors range from blue (negative loadings) to red (positive loadings), with intensity reflecting the magnitude of the loading. Average e': average early diastolic mitral annulus tissue velocity; LAVI: left atrial volume index; LVEF: left ventricular ejection fraction; LVMI: left ventricular mass index; LVVI: left ventricular volume index; Mitral A: mitral A-wave; Mitral DT: mitral deceleration time; Mitral E: mitral E-wave; PV time ratio: pulmonary vein time ratio; RA area: right atrial area; RWT: relative wall thickness; sPAP: systolic pulmonary artery pressure; TAPSE: tricuspid annular plane systolic excursion.

**Supplementary Figure 3-** Flow Chart of patients’ selection

|  |
| --- |

FUP: Follow-up; PVI: Pulmonary Vein Isolation.**Supplementary Figure 4-** AFEQT questionnaire scores

| **A**  **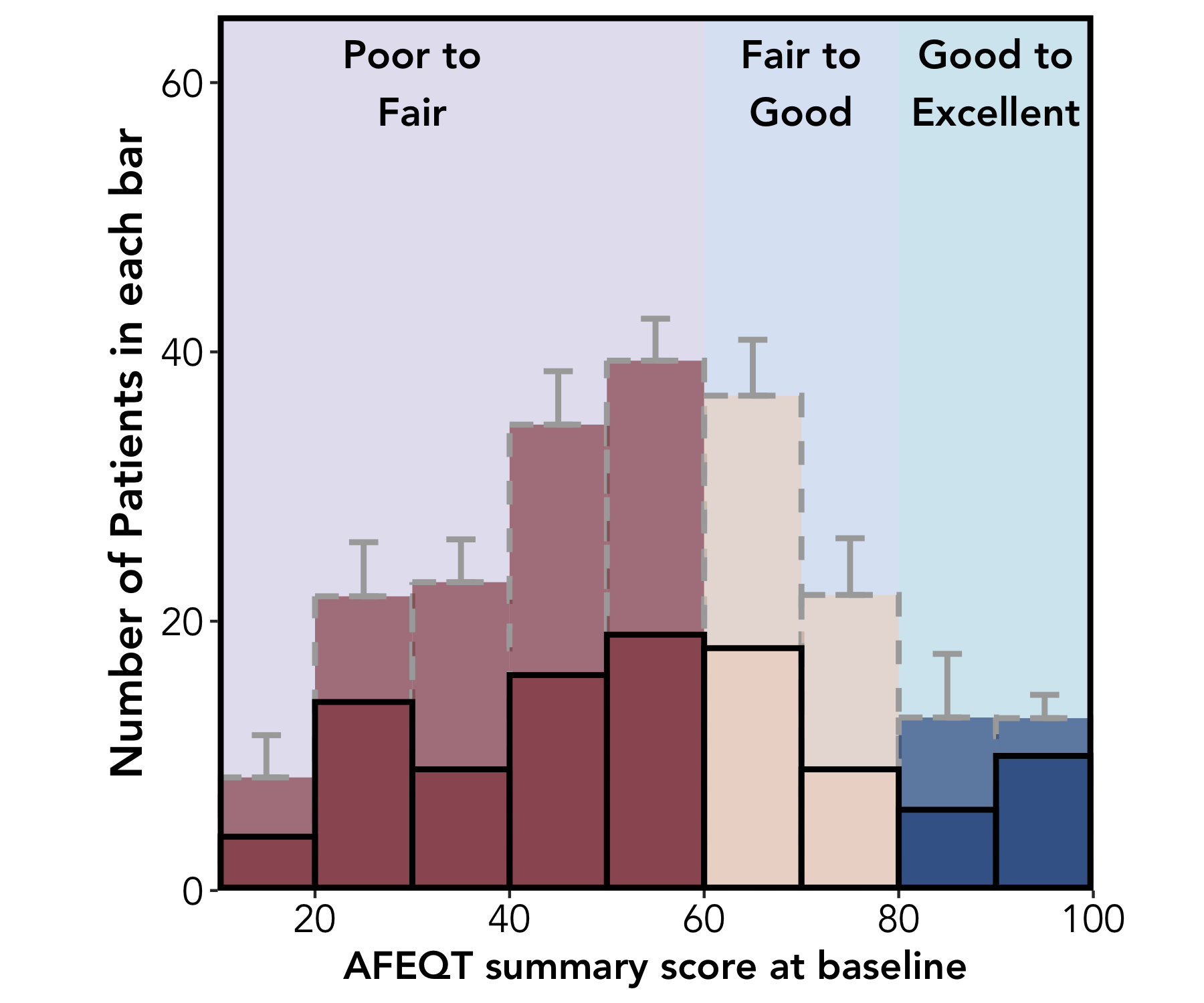** | **B**  **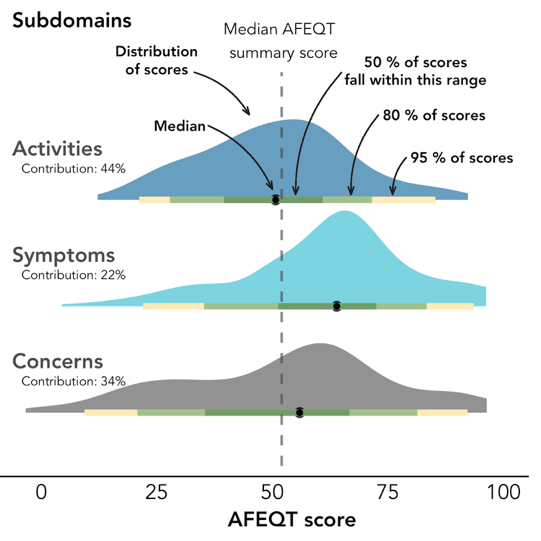** |
| --- | --- |

AFEQT questionnaire scores before **(A, B). (A)** Distribution of AFEQT summary scores at baseline among patients before AF ablation. The scores are categorized into Poor to Fair (0-60), Fair to Good (61-80), and Good to Excellent (81-100). The height of each **solid bar** represents the number of patients who completed the questionnaire. **Shaded bars** represent the estimated mean and standard deviation pooled across multiple imputations. **(B)** Half-eye plots for densities of baseline AFEQT scores across concerns, symptoms, and daily activities subdomains. The central black dots represent median values, while the shaded horizontal bars show multiple intervals.

**Supplementary Figure 5-** Choosing the optimal number of clusters (k)

| **A**  **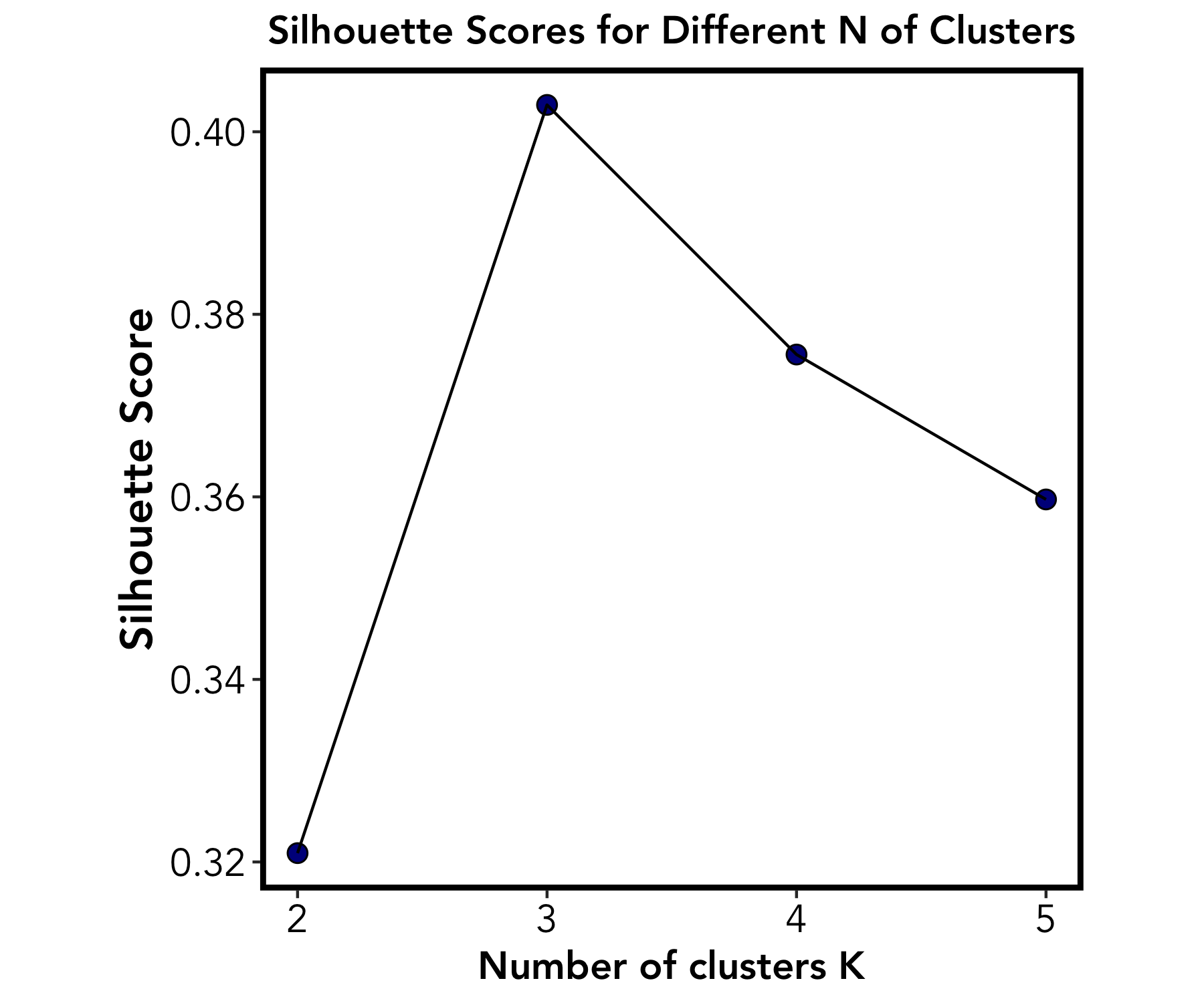** | **B**  **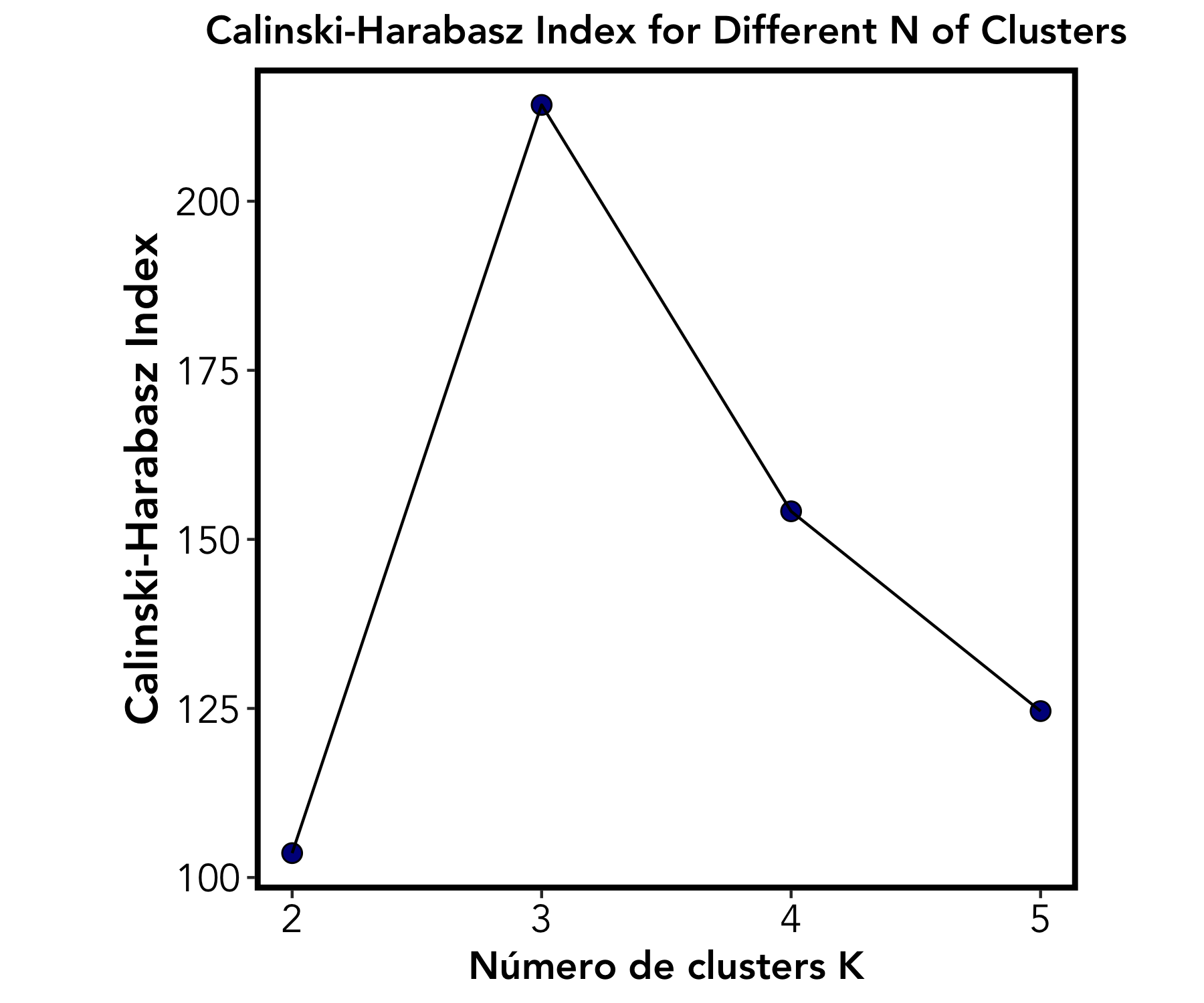** |
| --- | --- |
| **C**  **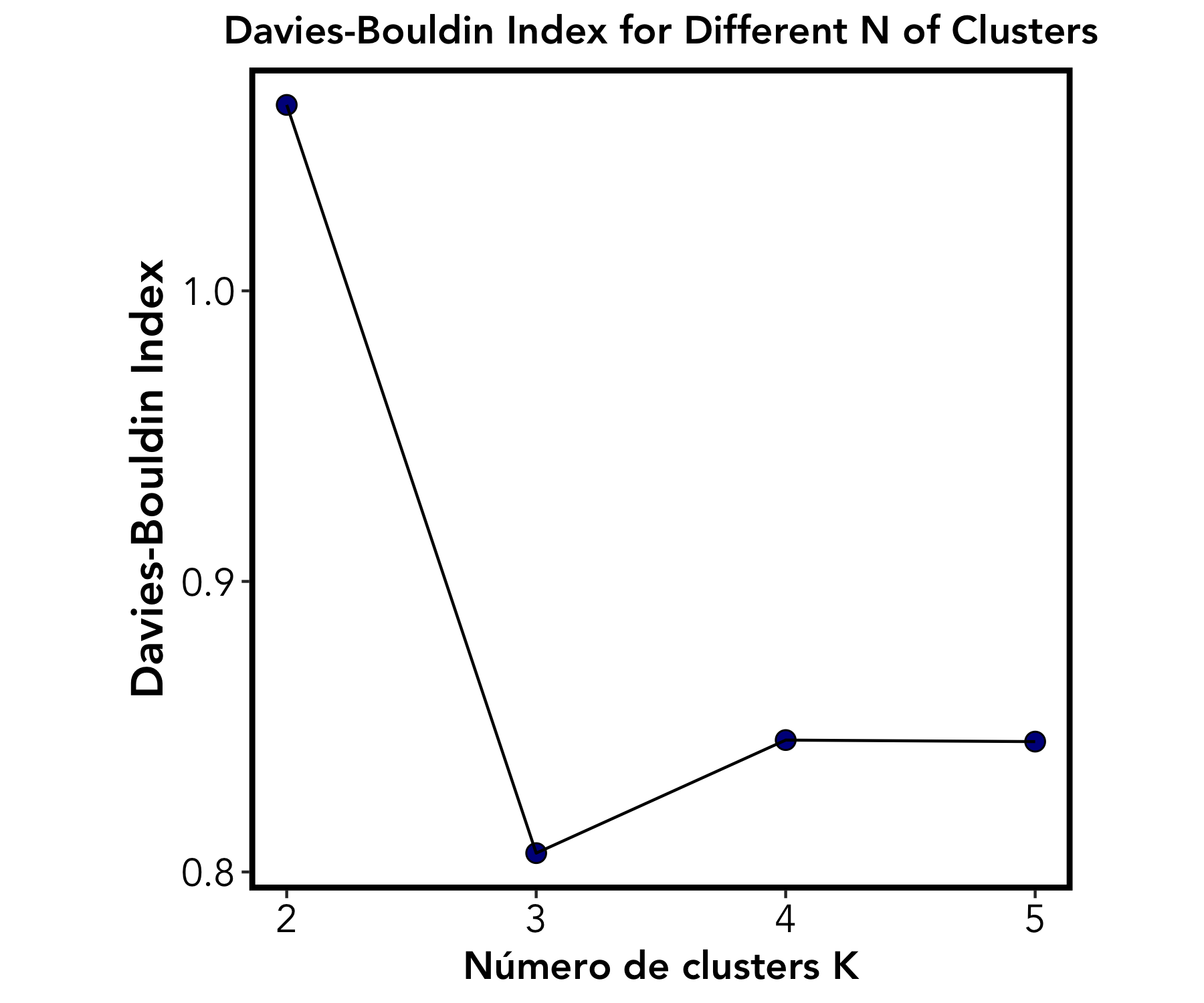** | **D**  **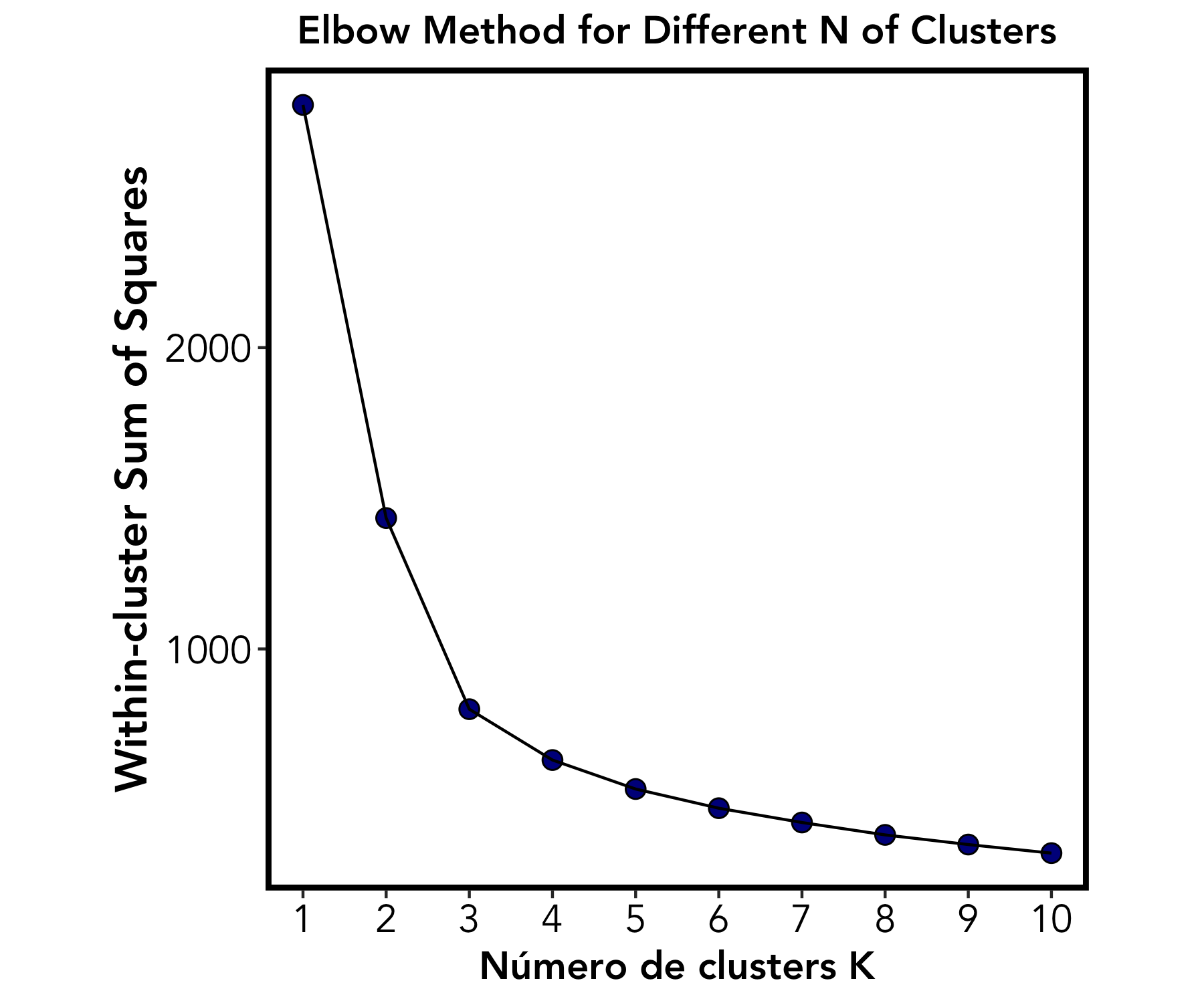** |
| **E**  **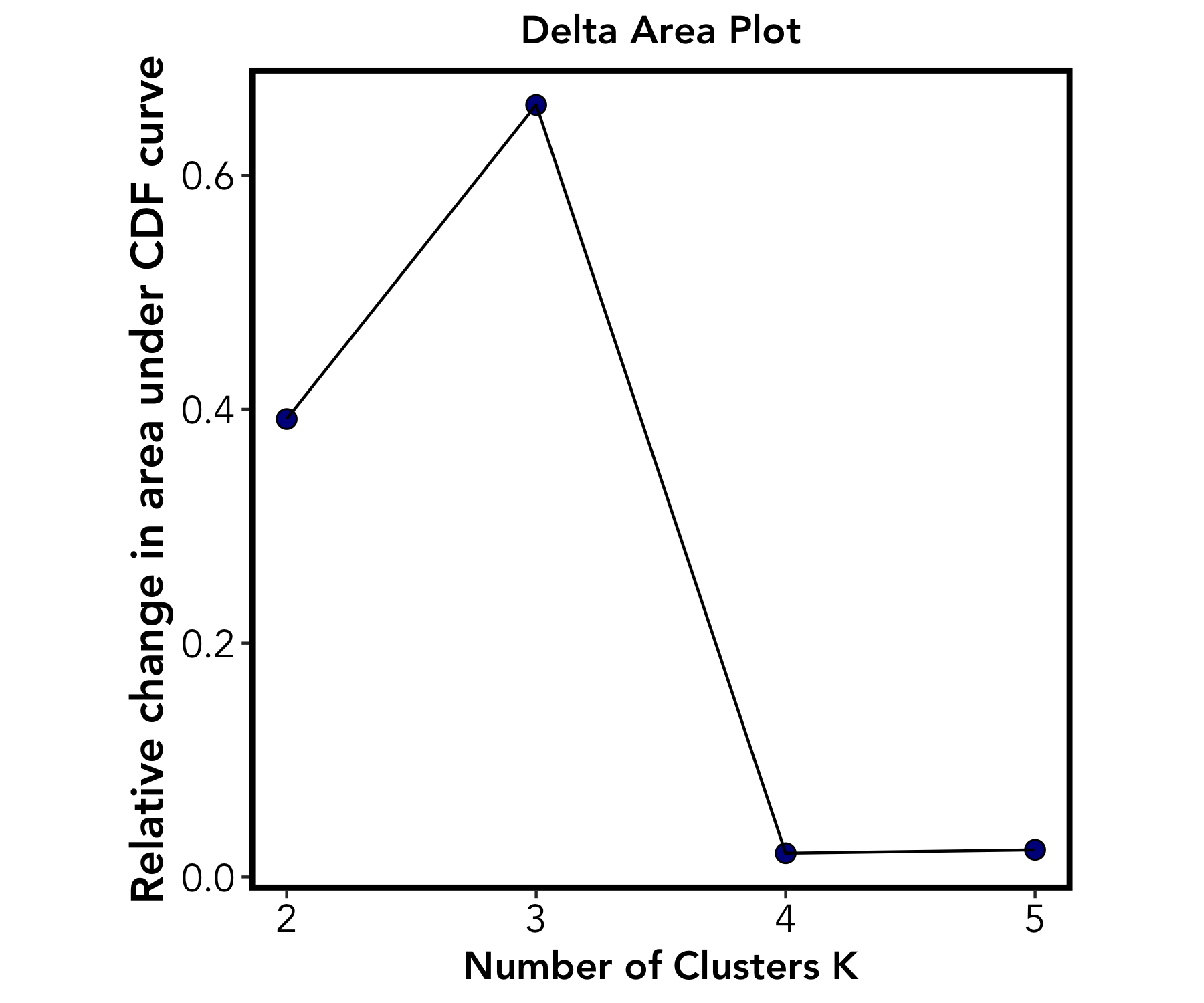** | **F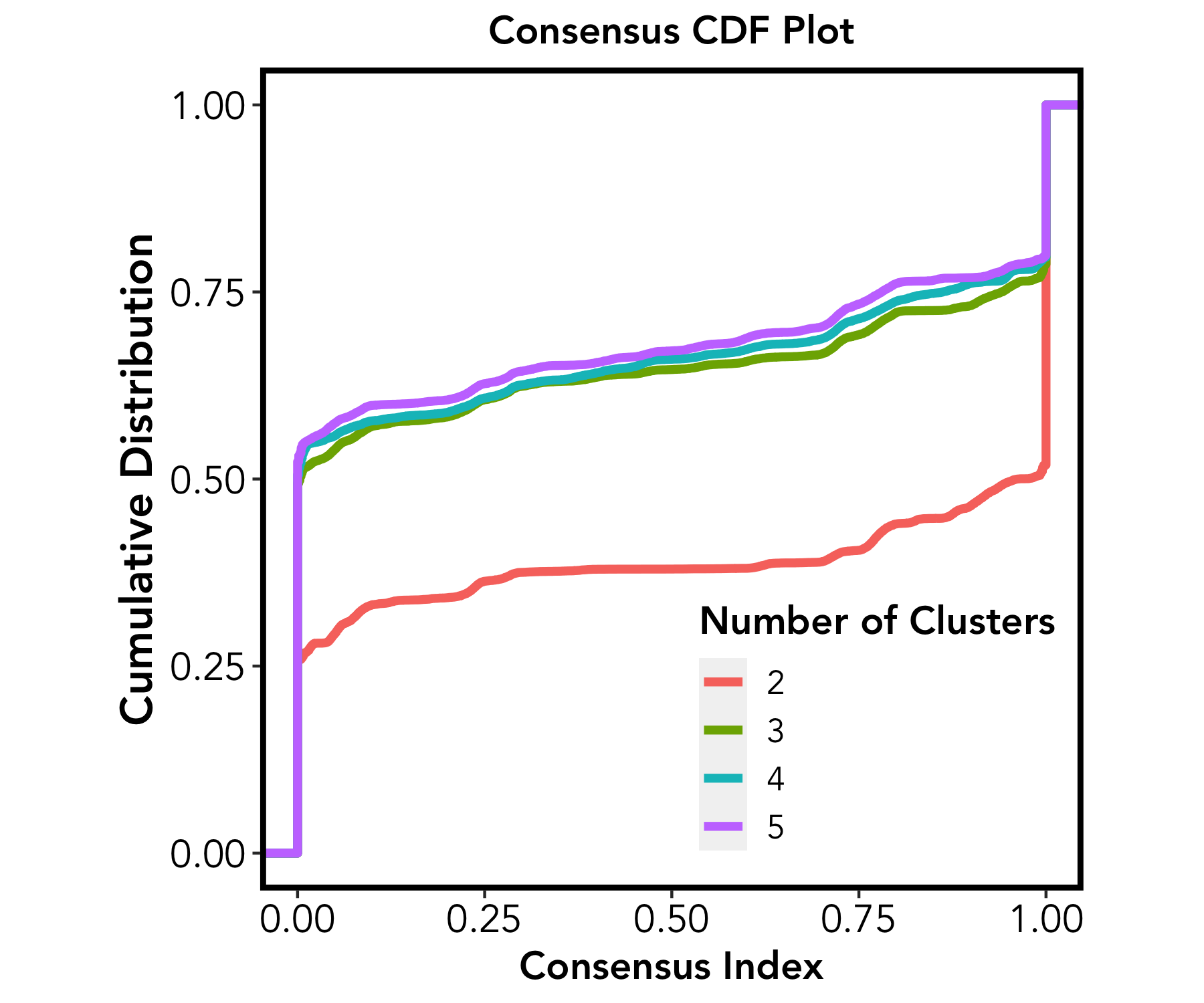** |
| **G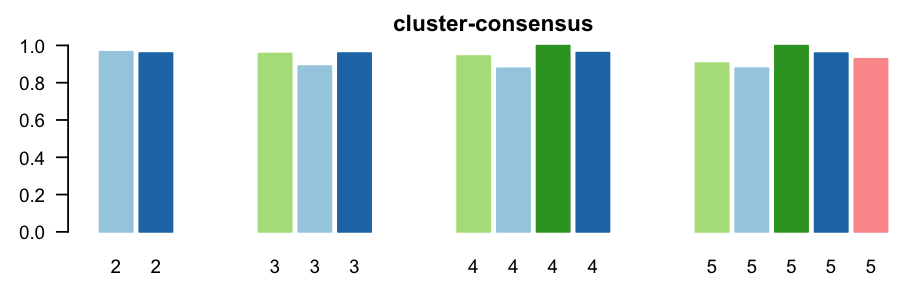** | |

**(A)** Silhouette Score - higher scores indicate better clustering quality. **(B)** Calinski-Harabasz Index - higher scores suggest a more optimal clustering solution. **(C)** Davies-Bouldin Index - lower scores indicate better clustering quality. **(D)** Elbow Method - the "elbow" point suggests an optimal k. (E) Delta Area Plot for Determining Optimal Number of Clusters- The plot shows a significant increase in the relative change when moving from 2 to 3 clusters, suggesting that 3 clusters may be the optimal number for capturing the most meaningful variance in the data. Beyond 3 clusters, the relative change stabilizes, indicating that additional clusters contribute minimally to model improvement. **(F)** Cumulative Distribution Function (CDF) Plot displays the CDF for each k to determine where the CDF reaches a maximum without compromising consensus. Higher and "flatter" curves are favorable. **(G)** Cluster-Consensus Plot shows the cluster-consensus values of clusters at each k. High values indicate cluster stability.

**Supplementary** **Figure 6-** Longitudinal trajectories of AFEQT overall summary scores by patient phenotype

**
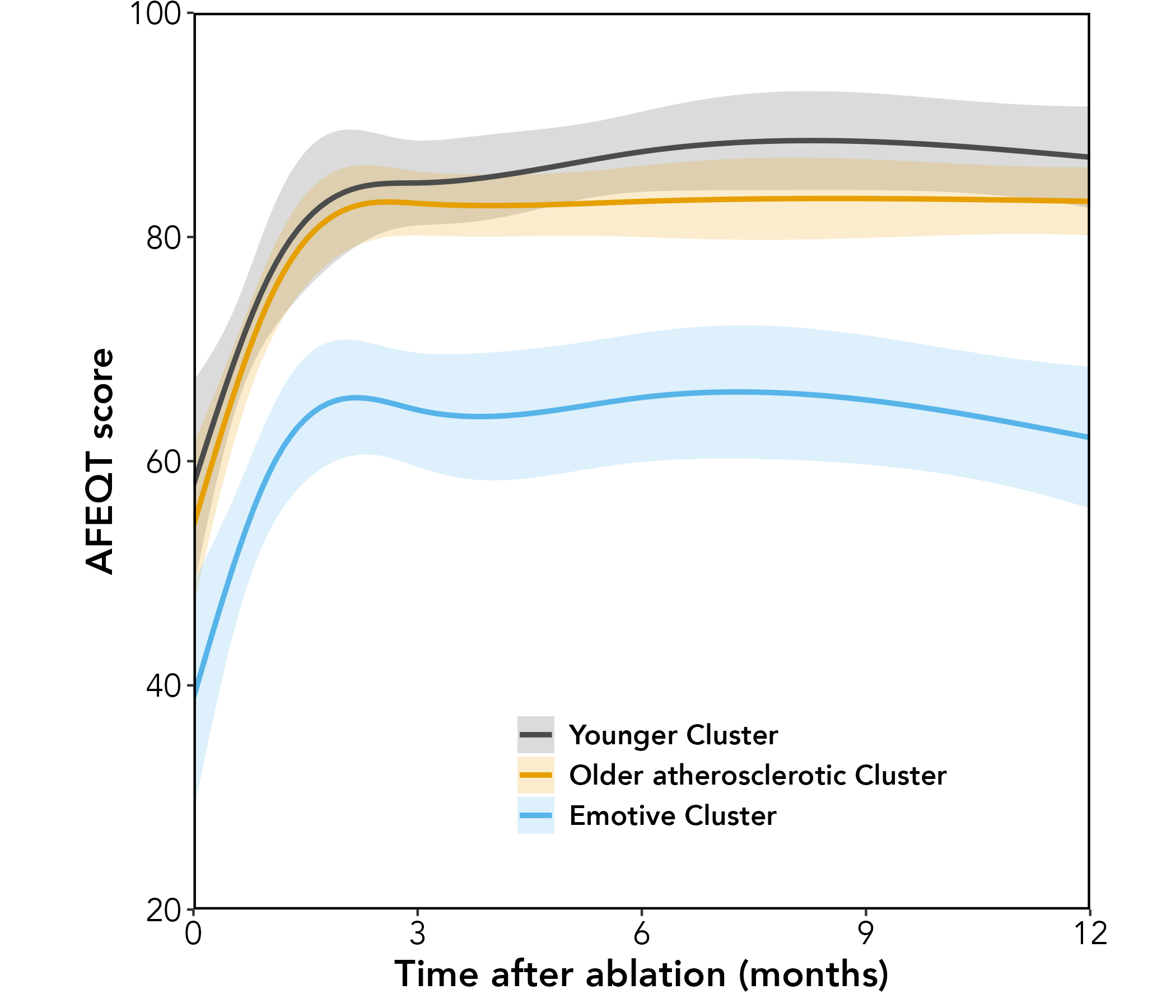
**

Longitudinal AFEQT summary score trajectories for the three patient phenotypes over 12 months post-ablation. Lines indicate estimated mean AFEQT-OS scores, while shaded areas depict their 95% confidence intervals

**Supplementary Figure 7-** Forest plot of rate ratios for total recurrences by patient cluster


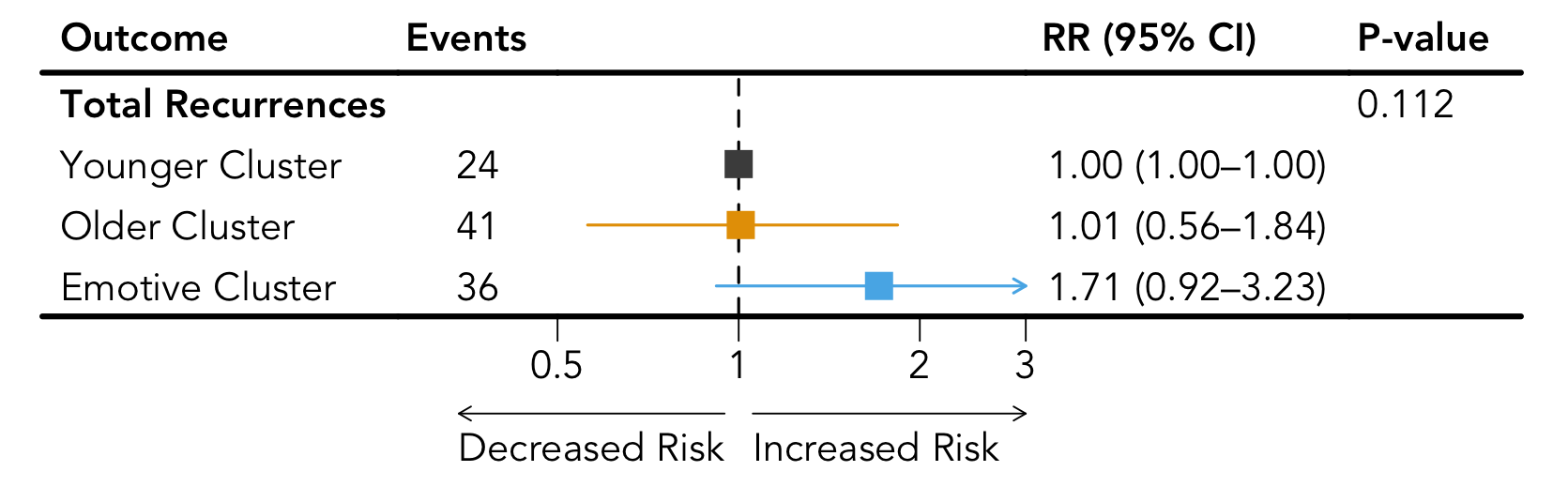


CI: Confidence interval; RR: Risk ratio

**References:**

1. Boehmke B, Greenwell B. *Hands-On Machine Learning with R*. 1st ed. Chapman and Hall/CRC, 2019.
